# Survival of *Escherichia coli* after high-antibiotic stress is dependent on both the pregrown physiological state and incubation conditions

**DOI:** 10.3389/fmicb.2023.1149978

**Published:** 2023-03-10

**Authors:** Lilja Brekke Thorfinnsdottir, Gaute Hovde Bø, James Alexander Booth, Per Bruheim

**Affiliations:** ^1^Department of Biotechnology and Food Science, Norwegian University of Science and Technology, Trondheim, Norway; ^2^Department of Microbiology, Oslo University Hospital, University of Oslo, Oslo, Norway

**Keywords:** persisters, antibiotics, metabolomics, metabolite profiling, *Escherichia coli*, relA, bioreactors

## Abstract

**Introduction:**

The survival of bacterial cells exposed to antibiotics depends on the mode of action, the antibiotics concentration, and the duration of treatment. However, it also depends on the physiological state of the cells and the environmental conditions. In addition, bacterial cultures contain sub-populations that can survive high antibiotic concentrations, so-called persisters. Research on persisters is challenging due to multiple mechanisms for their formation and low fractions, down to and below one millionth of the total cell population. Here, we present an improved version of the persister assay used to enumerate the amount of persisters in a cell population.

**Methods:**

The persister assay with high antibiotic stress exposure was performed at both growth supporting and non-supporting conditions. *Escherichia coli* cells were pregrown to various growth stages in shake flasks and bench-top bioreactors. In addition, the physiological state of *E. coli* before antibiotic treatment was determined by quantitative mass spectrometry-based metabolite profiling.

**Results:**

Survival of *E. coli* strongly depended on whether the persister assay medium supported growth or not. The results were also highly dependent on the type of antibiotic and pregrown physiological state of the cells. Therefore, applying the same conditions is critical for consistent and comparable results. No direct connection was observed between antibiotic efficacy to the metabolic state. This also includes the energetic state (i.e., the intracellular concentration of ATP and the adenylate energy charge), which has earlier been hypothesized to be decisive for persister formation.

**Discussion:**

The study provides guides and suggestions for the design of future experimentation in the research fields of persisters and antibiotic tolerance.

## 1. Introduction

Antimicrobial resistance (AMR) is increasing and is considered a global health threat. A recent comprehensive review estimates that more than 6 million deaths worldwide were related to AMR in 2019 (Murray et al., 2022). During the last decades, few new antibiotics have been discovered, and there is an urgent need to develop new antibiotics to tackle the increasing resistance (Lewis, 2020). However, it is also imperative to study the bacterial stress responses to known and already approved antibiotics to optimize and develop new clinical intervention strategies (Meylan et al., 2018; Murray et al., 2022; Verstraete et al., 2022). Bacteria have evolved advanced and diverse strategies for survival during environmental, nutritional, and toxic stress conditions, including antibiotic exposure (Hengge, 2011; Tkachenko, 2018). New targets for antimicrobial therapy can also be identified through such research. Many basic studies relate the applied concentration of antibiotics to either a fraction or multiple of the minimal inhibitory concentration (MIC). MIC is recorded by a standardized protocol (Andrews, 2001) and is not directly relevant to stress response and physiological studies in growing cultures at different densities. Nevertheless, it is a relevant measure for estimating high-and low-stress exposures. Low-stress exposure studies aim to monitor stress responses at high survival rates, while high-exposure studies aim to assess killing effects.

Interestingly, already in the 1940s, it was realized that bacterial populations contain a subpopulation that is tolerant to high doses (> 10–100 × MIC) of antibiotics, called persisters or persistent bacteria (Hobby et al., 1942; Bigger, 1944). This subpopulation is genetically identical to their sensitive counterparts, unlike antibiotic-resistant bacteria that have acquired mutations that increase their tolerance (Balaban et al., 2019). Persisters are believed to contribute to recalcitrant infections and increase the likelihood of antibiotic resistance development (Levin-Reisman et al., 2017). Persister cells arise either spontaneously or are triggered by different environmental stimuli. Spontaneous persisters occur with very low frequencies (e.g., < 10^−3^ to 10^−6^) in normal growing cultures (Balaban et al., 2019), which makes revealing the mechanisms behind their formation challenging. Studying the metabolic state of the cells activating and reaching this survival state is also challenging due to the low frequency of formation. Recent single-cell studies have provided some novel insights, and future developments in such analytical methodology will be crucial for further advances (Shan et al., 2017; Kim et al., 2018; Svenningsen et al., 2019; Manuse et al., 2021). Triggered persisters often occur with higher frequencies, and the environmental stimuli that can induce these include starvation, changes in pH, high cell numbers, and exposure to antibiotics (Balaban et al., 2019).

Persistence triggered by antibiotic exposure has a clear clinical relevance regarding recurrent infections and the potential development of antibiotic resistance (Balaban et al., 2019; Windels et al., 2019). Nutritionally triggered persistence is also an essential adaptation to survive starvation and harsh conditions (Brown, 2019), not for the individual bacteria but the bacterial community as a whole, like a bet-hedging strategy. Toxin–antitoxin systems and the stringent response signaling molecule guanosine pentaphosphate [(p)ppGpp] have been associated with triggered persistence (Chowdhury et al., 2016; Goormaghtigh et al., 2018; Holden and Errington, 2018; Svenningsen et al., 2019; Pacios et al., 2020). Despite this, no consensus on the mechanism of formation has been reached, and there are probably several events or mechanisms that promote survival upon exposure to high doses of antibiotics.

Since the exact mechanisms for persister formation and resuscitation remain to be fully elucidated, further studies on different aspects of these survivors of high doses of antibiotics are needed. The research field of bacterial persistence has struggled with many controversies due to ambiguous definitions and the fact that there is likely not one single, unique mechanism associated with the formation of persisters (Wilmaerts et al., 2019). Contradictory reports in the literature need to be evaluated with close attention to the experimental design (Kim and Wood, 2016, 2017; Harms et al., 2017). Therefore, it was highly pertinent that many contributors in this field came together and discussed definitions and guidelines for research on antibiotic persistence a few years ago (Balaban et al., 2019). Despite this, the protocols and study designs reported in the persister literature still show significant differences, making comparisons between studies challenging.

The experimental setup used to investigate survival after high doses of antibiotics is often referred to as the persister assay. This is usually performed by transferring aliquots of whole cultures to vials before treatment with high doses of antibiotics (10 – 100 × the MIC; Goormaghtigh and Van Melderen, 2016). The surviving persisters are then monitored by use of colony-forming unit (CFU)-plating on growth-supporting solid agar. The CFU-plating is often performed in time series after treatment to observe the characteristic biphasic killing curve, termed the hallmark of persisters (Goormaghtigh and Van Melderen, 2016; Balaban et al., 2019). The surviving fraction after the persister assay will regrow in conditions without antibiotics and display the same frequency of persisters upon subsequent antibiotic exposure, unlike resistant bacteria that would have an increased survival upon subsequent exposures (Balaban et al., 2019). We have revisited two critical stages in the bacterial responses to high doses of antibiotics: the pre-cultivation conditions before antibiotic treatment and the nutritional conditions during treatment. Reproducible experimental conditions, such as cultivation, are prerequisites for unambiguous data. First, using a defined mineral growth medium instead of complex, rich medium (such as lysogeny broth, LB) makes the cultivations, and hence results, more reproducible, which has previously been discussed (Harms et al., 2017). Using defined medium also enables the design of conditions with clearly defined nutrient limitations, such that the cells run out of a specific nutrient first (for example, nitrogen or carbon). In contrast, nutritional depletion is difficult to follow in rich medium since it has several possible carbon and nitrogen sources and continuously runs out of amino acids during cultivation. Secondly, bioreactors are highly recommended since pH and oxygen level can be monitored and controlled continuously.

In the persister assay, both media and bacterial density will differ when assaying cultures from different growth states or time points after inoculation or nutritional stress. In this study, we show the importance of performing antibiotic treatment for persister assays in the same medium background to obtain valid comparisons of surviving frequencies for different growth states. We investigate the survival of spontaneous and triggered *Escherichia coli* persisters and have chosen to use the broader term surviving fraction. Our high-antibiotic stress (HAS) assay is similar to the conventional persister assay but has defined and comparable conditions during HAS. We included three antibiotics with different modes of action; ciprofloxacin, ampicillin, and streptomycin, which are commonly used in the field (examples: Dörr et al., 2010; Chowdhury et al., 2016; Harms et al., 2017). Finally, we undertook a thorough metabolite profiling of the central carbon metabolism to unravel any potential link between the metabolic state of the whole culture before HAS and the surviving fraction in the different conditions.

## 2. Materials and methods

### 2.1. Cultivation, strains, and media

*Escherichia coli* K-12 MG1655 (700926^™^, ATCC^®^) and a *ΔrelA* mutant of this strain were used for all experiments. The *∆relA* strain was generated by creating a P1 lysate from the Keio strain JW2755-3 (Baba et al., 2006) and transducing this into the above *E. coli* strain, and selecting with kanamycin (50 μg/ml). The kanamycin resistance selection marker, flanked by FRT sites, was removed using the FLP recombinase containing plasmid pCP20 (Datsenko and Wanner, 2000).

For all experiments, overnight cultures (ONC) were prepared by adding 100 μl of a glycerol freeze stock (20% glycerol, stored at –80°C) of the bacterial strain to 100 ml glucose mineral medium in a 500 ml baffled shake flask. The cultures were incubated at 37°C with continuous stirring at 200 rpm for 16 ± 1 h.

#### 2.1.1. Shake flask medium

The glucose mineral medium for shake flasks was prepared in milliQ-H_2_O (18.2 MΩ cm) by dissolving 11.2 g/l Na_2_HPO_4_–7H_2_O (S9390, Sigma-Aldrich), 3 g/l KH_2_PO_4_ (P5655, Sigma-Aldrich), 0.5 g/l NaCl (27810.295, VWR), 1 g/l NH_4_Cl (A9434, Sigma-Aldrich), 0.2465 g/l MgSO_4_–7H_2_O (M5921, Sigma-Aldrich), 4 g/l glucose (101176 K, VWR), 2 ml/l of a 50 mg/l CoCl_2_–6H_2_O solution (C8661, Sigma-Aldrich) and 2 ml/l medium of a trace element solution containing 10 g/l FeSO_4_–7H_2_O (F8633, Sigma-Aldrich), 2.25 g/l ZnSO_4_–7H_2_O (Z0251, Sigma-Aldrich), 2 g/l CaCl_2_–2H_2_O (223506, Sigma-Aldrich), 1 g/l CuSO_4_–5H_2_O (197722500, Fisher Scientific), 0.38 g/l MnCl_2_–4H_2_O (M5005, Sigma-Aldrich), 0.14 g/l H_2_BO_3_ (B6768, Sigma-Aldrich) and 0.1 g/l (NH_4_)6Mo_7_O_24_–4H_2_O (1011820250, Merck).

#### 2.1.2. Bioreactor media and cultivations

One liter stirred benchtop Applikon bioreactors were used for all bioreactor experiments, with the same instrumental setup as described in (Thorfinnsdottir et al., 2023). The temperature was kept constant at 37°C, and pH was monitored and adjusted automatically by addition of 4 M NaOH (28244.295, VWR). Aeration was ensured by continuous sparging of 600 ml/min of air and automatic increase of stirring to keep the level of dissolved oxygen above 30%. The bioreactor medium is described in Thorfinnsdottir et al. (2023). This medium contained 5 g/l NH_4_Cl and 10 g/l glucose as nitrogen and carbon source, respectively, and was used for exponential phase and carbon-limitation experiments. For nitrogen-limitation experiments, the amount of NH_4_Cl and glucose was changed to 1.7 and 20 g/l, respectively. Bacterial growth was monitored by both optical density measurements at 600 nm (OD_600_) and by continuous monitoring of CO_2_ production and O_2_ usage. Entry to stationary phase was defined as a sudden and sharp decrease in the CO_2_ level in the exhaust gas (and a corresponding increase in O_2_ level), combined with a reduction of stirring rate due to reduced oxygen consumption (see Supplementary Figure 1 for data from a representative cultivation).

#### 2.1.3. Preparation of solid medium

Solid medium was made with 10 g/l tryptone (T9410, Sigma-Aldrich), 5 g/l yeast extract (92144, Sigma-Aldrich), 5 g/l NaCl and 15 g/l bacteriological agar (LP0011, ThermoFisher Scientific^™^) in distilled water, and autoclaved. The agar was spread on Petri dishes (82.1473.001, Sarstedt) and left upside down until use for CFU measurements.

### 2.2. Determination of minimal inhibitory concentrations

Determination of minimal inhibitory concentrations (MIC) of the three antibiotics, ampicillin (A9393, Sigma-Aldrich), ciprofloxacin (17850, Sigma-Aldrich), and streptomycin (S6501, Sigma-Aldrich), were performed 96-well plates, with two-fold dilutions of the antibiotics, according to (Andrews, 2001). An ONC was diluted with fresh media to OD_600_ 0.1, and further diluted 1:100. The bacterial suspension was further diluted 1:2 in the wells to a total volume of 150 μl in each well, including antibiotics. The plates were incubated with shaking (37°C, 600 rpm), and absorbance was read after 24 h (Tecan Spark^®^ 20 M). MIC was determined to the lowest concentration without any visible growth.

### 2.3. High-antibiotic stress experiments

#### 2.3.1. Preliminary experiments in shake flask

An ONC (*E. coli* WT) was diluted 1:200 into 100 ml fresh glucose mineral medium in a shake flask and incubated as the ONC until the OD_600_ reached 0.5 (approximately 6 h). Aliquots (12 ml) of the culture were centrifuged and resuspended in media (12 ml) with different compositions, and antibiotics were added. The different media were similar to the shake flask media, only without glucose and/or NH_4_Cl. The final concentrations were 100 μg/ml ampicillin or 5 μg/ml ciprofloxacin (17850, Sigma-Aldrich). Each aliquot was then distributed to three 13 ml test tubes (62.515.006, Sarstedt) with 2 l in each tube and incubated at 37°C with shaking at 300 rpm. The lid of each tube was not completely closed, and the lids were kept at the same place for all replicas to ensure equal aeration of the cultures, as suggested by Harms et al. (2017). A sample was taken for CFU-plating before antibiotic treatment to measure the initial CFU/mL, and after 5 h of incubation. The aliquots were centrifuged (5 min, 4500 *g*) and resuspended in 0.85% NaCl to remove the antibiotics. A ten-fold serial dilution was performed, and 100 μl of appropriate dilutions were spread on LB agar plates. The plates were incubated at 37°C for approximately 20 h before colonies were counted. The surviving fractions were calculated by dividing the CFU/mL at 5 h by the initial CFU/mL.

#### 2.3.2. High-antibiotic stress experiments in bioreactors

An ONC of either *E. coli* WT or *ΔrelA* was inoculated into a bioreactor. After inoculation, the total volume in the bioreactors was 900 ml, and the inoculum volume was 1%. Aliquots were sampled for HAS at OD_600_ = 3 for the exponential phase or 20 min after entry into stationary phase (due to either carbon or nitrogen limitation). The different sample aliquots were centrifuged and resuspended with dilution to OD_600_ = 0.5 in either complete or nitrogen-free (N-free) media, with or without antibiotics. The final concentrations were 100 μg/ml ampicillin, 5 μg/ml ciprofloxacin, or 25 μg/ml streptomycin. Each sample was then distributed into three 13 ml replicate test tubes. Incubation and CFU-plating were as described for the shake flask experiments.

### 2.4. Metabolomics

Absolute quantitative MS-based metabolite profiling was performed on the bulk culture of *E. coli* WT and the *ΔrelA* mutant in the bioreactor at the same time points as samples were taken for HAS experiments (OD600 = 3 in exponential phase and 20 min after entry into nitrogen-limited stationary phase). Additionally, metabolite profiling was performed from the cultures immediately after centrifugation and resuspension into complete or N-free media, but this time without antibiotics. Sampling for metabolite profiling was performed by fast filtration, and both sampling and sample processing are described in (Thorfinnsdottir et al., 2023). The analysis of phosphorylated metabolites is described in Kvitvang et al. (2014) with modifications in Stafsnes et al. (2018), and organic acids and amino acids were analyzed as described in Røst et al. (2020). Adenylate energy charge (AEC) was calculated from absolute intracellular concentrations of AMP, ADP, and ATP by the following equation ([ATP]*0.5[ADP])/([ATP] + [ADP] + [AMP]) (Atkinson and Walton, 1967).

### 2.5. Statistical analysis

#### 2.5.1. Statistical analysis for high-antibiotic stress experiments

**Figure 1:**
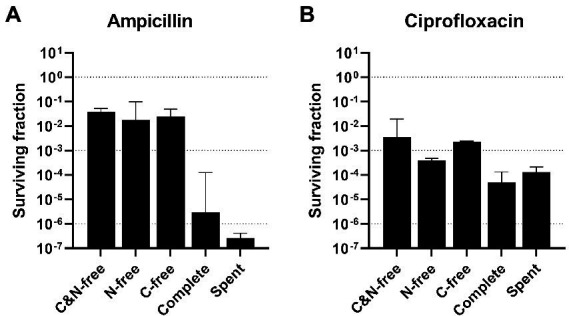
Surviving fractions after high-antibiotic stress (HAS) in different media. Aliquots of mid-exponential phase *Escherichia coli* WT at OD600 = 0.5 in shake flasks were centrifuged and resuspended in different stress media (without either carbon or nitrogen source or both, complete, or spent) and treated with **(A)** 100 μg/ml ampicillin or **(B)** 5 μg/ml ciprofloxacin for 5 h. Viable cell counts were assayed at 0 and 5 h after treatment, and the surviving fractions at 5 h were calculated. Geometric mean and geometric standard deviation from 2–4 experiments are plotted, with a dotted line at 10^0^, 10^−3^, and 10^−6^ to aid interpretation. HAS, high-antibiotic stress; C&N-free, lacking both carbon and nitrogen; N-free, lacking nitrogen; C-free, lacking carbon; spent, supernatant from mid-exponential phase cultures.

**Figure 2:**
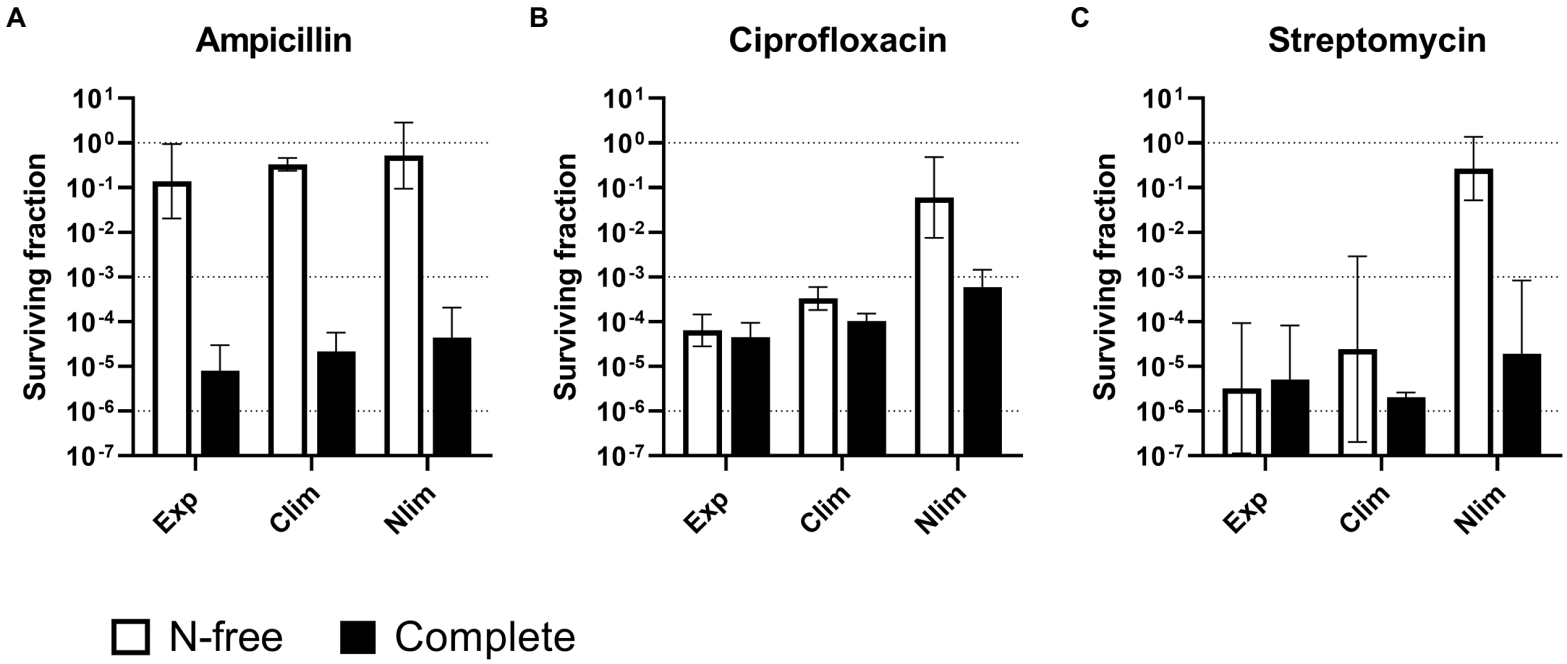
Survival of *Escherichia coli* WT cells pregrown to three physiological states and treated with antibiotics in two stress media. *Escherichia coli* WT cells were pregrown to three physiological states in benchtop bioreactors before centrifugation and resuspension into two stress media with **(A)** 100 μg/ml ampicillin, **(B)** 5 μg/ml ciprofloxacin or **(C)** 25 μg/ml streptomycin. Viable cell counts were assayed at 0 and 5 h after treatment, and the surviving fractions at 5 h were calculated. Geometric mean and geometric standard deviation from 2 to 4 experiments are plotted, with a dotted line at 10^0^, 10^−3^, and 10^−6^ to aid interpretation. N-free, HAS medium lacking nitrogen; Exp, exponential phase; Clim, carbon-limited stationary phase; Nlim, nitrogen-limited stationary phase.

**Figure 3:**
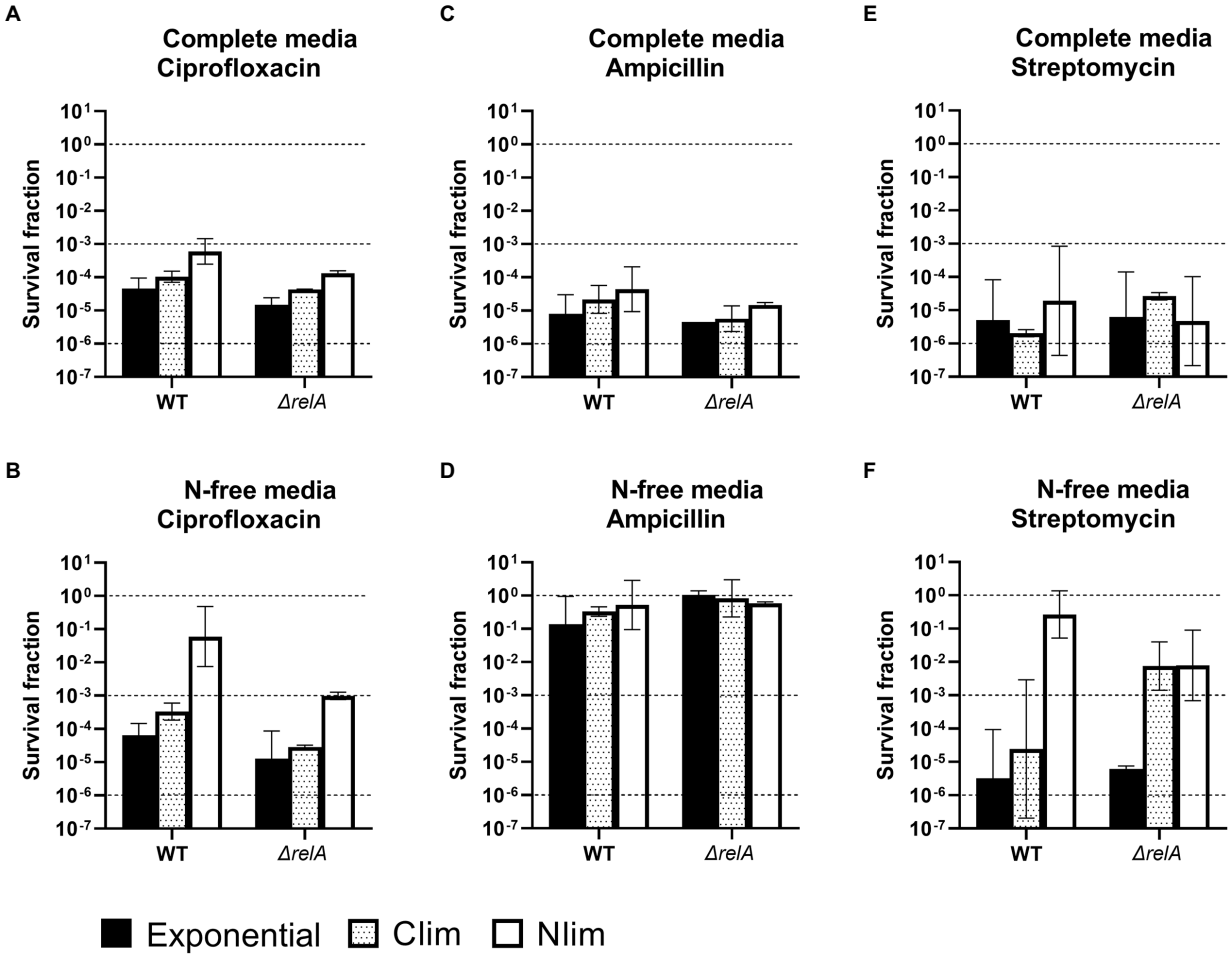
Survival of *E. coli* WT and *ΔrelA* cells. *E. coli* WT and *ΔrelA* cells were pregrown to three physiological states (exponential phase, carbon or nitrogen-limited stationary phase; Clim or Nlim) in benchtop bioreactors, before centrifugation and resuspension into two stress media with **(A)** and **(B)** 5 μg/ml ciprofloxacin, **(C)** and **(D)** 100 μg/ml ampicillin or **(E)** and **(F)** 25 μg/ml streptomycin. Viable cell counts were assayed at 0 and 5 h after treatment, and the surviving fractions at 5 h were calculated. Geometric mean and geometric standard deviation from 2 to 4 experiments are plotted, with a dotted line at 10^0^, 10^−3^, and 10^−6^ to for visualization. WT results from Figure 2 are included to ease comparison and interpretation. N-free, HAS medium lacking nitrogen; Exp, exponential phase; Clim, carbon-limited stationary phase; Nlim, nitrogen-limited stationary phase.

Surviving fractions were calculated by dividing the geometric mean of triplicate CFU measurements from each time point by the geometric mean of triplicate CFU measurements from 0 h before antibiotic treatment (Goormaghtigh and Van Melderen, 2016). The geometric mean and standard deviations of surviving fractions are presented in Figures 1–3.

#### 2.5.2. Statistical analysis for metabolome data

**Figure 4:**
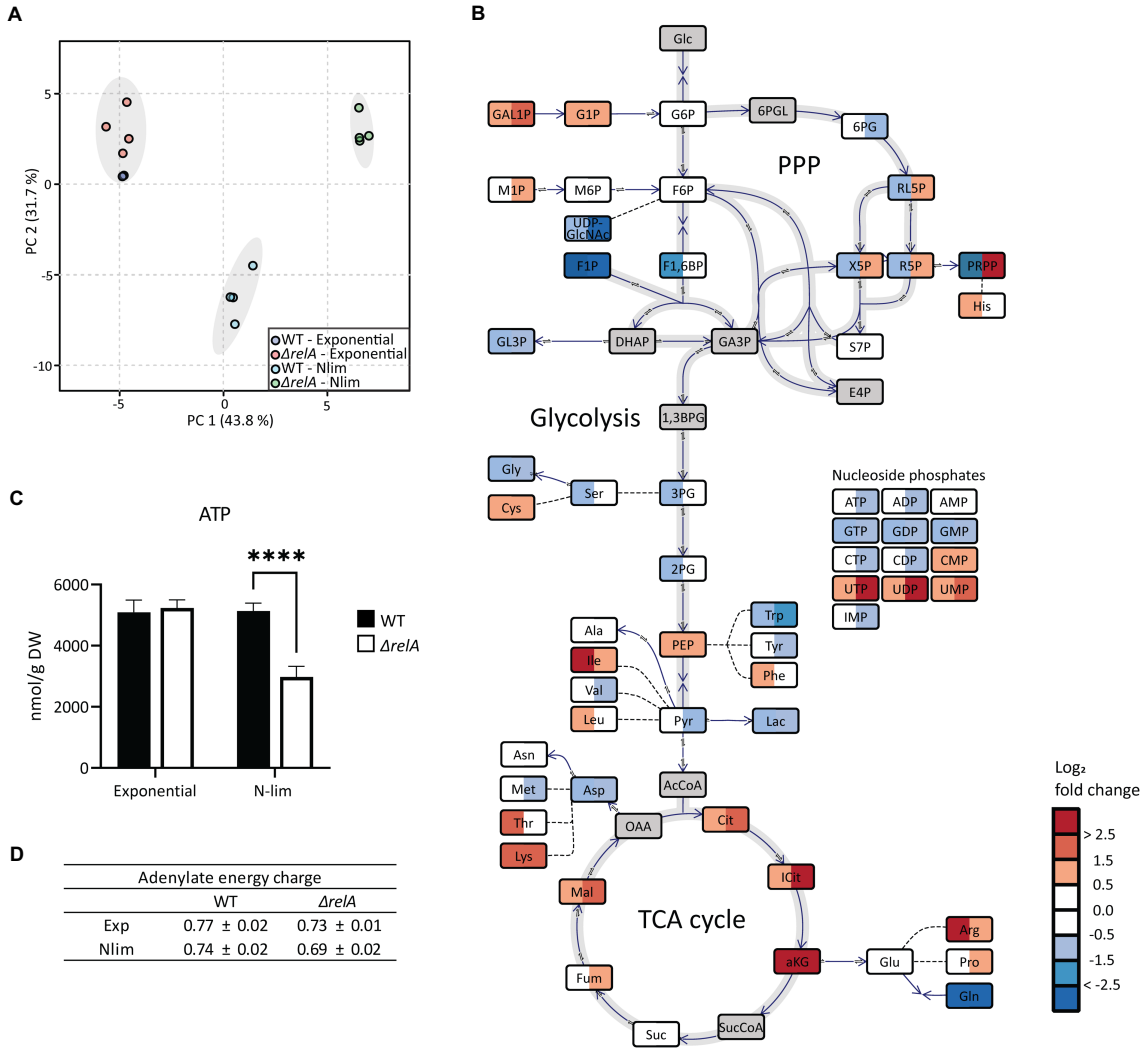
Metabolite profiling of *E. coli* WT and *ΔrelA* in both exponential and nitrogen-limited stationary phases. Samples for metabolite profiling were taken directly from the bioreactor in exponential phase at OD600 = 3 or 20 min after entry to Nlim stationary phase. **(A)** Principal component analysis with 57 metabolites was performed with all samples, **(B)** log2 fold change in Nlim stationary phase relative to exponential phase. WT is to the left, *ΔrelA* to the right in the boxes. Grey indicates not measured. The concentrations used to calculate the ratios are presented in Supplementary Table 1. **(C)** Absolute ATP concentrations, with unpaired, two-tailed *t*-test between each condition, *n* = 4. ^*^p ≤ 0.05, ^**^p ≤ 0.01, ^***^p ≤ 0.001. **(D)** Adenylate energy charge (AEC), calculated using the absolute concentrations of ATP, ADP, and AMP, with *n* = 4. Metabolite abbreviations are presented in Supplementary Table 2.

The processing and analysis of metabolome data were performed as described in (Thorfinnsdottir et al., 2023). Normalization was performed to dry weight (DW), interpolated from a corresponding g DW/l versus OD_600_ curve. Principal component analyses (PCA) and *t*-tests were performed with MetaboAnalyst 5.0 (Chong et al., 2019), and the central metabolism plot in Figure 4 was made with OMIX^®^ (Droste et al., 2013). Standard deviations for AEC were calculated using error propagation.

## 3. Results

### 3.1. Survival after high-antibiotic stress is dependent on medium composition

Previous studies have mostly treated growing cultures by adding antibiotics directly into the growth media, often in rich media, as described for the persister assay (Harms et al., 2017). By doing this, survival during various growth conditions, such as pH, oxygenation level, or media composition has been compared. Therefore, preliminary experiments were performed to explore the dependency of survival on the media composition during stress. Aliquots of mid-exponential phase *E. coli* WT at OD_600_ = 0.5 from shake flask cultures were centrifuged and resuspended in different media (referred to as stress media to distinguish it from the initial growth media) with high doses of ampicillin or ciprofloxacin. We refer to this as the High-Antibiotic Stress (HAS) assay. Surviving fractions after 5 h were measured, as this, in our experience, is a suitable time when the biphasic killing curve has flattened (see Supplementary Figure 2 for representative biphasic killing curves). The MIC for ampicillin and ciprofloxacin were determined to 1.6 and 0.01 μg/ml, respectively. The concentrations of ampicillin and ciprofloxacin for HAS are commonly used in persister assays in the literature (examples: Dörr et al., 2010; Chowdhury et al., 2016; Harms et al., 2017), and are more than 20 × the MIC of the respective antibiotic for our conditions.

After the HAS assay with ampicillin, surviving fractions were four orders of magnitude higher when the stress medium lacked some components essential for growth, compared with the complete growth medium (Figure 1A; Supplementary Figure 3A). There were small differences, less than one order of magnitude, between the three growth-deficient stress media (lacking either carbon or nitrogen sources or both). The spent medium is the supernatant from the mid-exponential (OD_600_ = 0.5) shake flask cultures and, therefore, still has growth potential. Thus, the spent medium would be expected to result in a similar surviving fraction as the complete medium. However, the lower survival in the spent media might indicate release of components to the medium during growth that increases stress sensitization. This could be proteases from cell lysis or cell wall fragments such as muropeptides, known for various functions (Irazoki et al., 2019).

The same trend was observed for the HAS assay with ciprofloxacin, but with far smaller differences between the growth deficient and the growth supporting stress media, only 1–2 orders of magnitude (Figure 1B; Supplementary Figure 3B). These results demonstrate the limitations of the traditional persister assay, as the surviving fraction strongly depends on the nutritional availability during treatment. Therefore, if cultures that are pregrown to different physiological states and hence different media compositions are to be compared, biases introduced by the original cultivation medium background need to be removed. These preliminary results motivated us to perform a more thorough investigation of HAS survival under conditions independent of the composition of the cultivation medium.

### 3.2. Survival in N-free stress medium is dependent on the pregrown physiological state

The subsequent experiments were performed with *E. coli* pregrown to different growth states in benchtop bioreactors since those provide better control of the pregrown state. Continuous sparging of air into the bioreactor and increased stirring in response to reduced levels of dissolved oxygen prevent oxygen limitation at any time during the cultivation. The pH is also continuously monitored and controlled. Continuous logging of respiratory data from measurements of exhaust gas composition also enables close monitoring of the growth stages and reproducible sampling time (see an example of data from bioreactor experiments in Supplementary Figure 1). HAS survival was investigated for *E. coli* WT cells pregrown to three physiological states (exponential and either carbon or nitrogen-limited stationary phase, Clim and Nlim, respectively), with two stress media (complete or nitrogen-freem N-free), and three antibiotics with different modes of action (ampicillin, ciprofloxacin, and streptomycin). The concentration of streptomycin were also based on literature (Dörr et al., 2010), and on being more than 20 × the MIC in our conditions (MIC determined to 1.25 μg/ml).

The HAS survival with ampicillin entirely depended on the stress medium, not the pregrown physiological state. For all pregrown states, ampicillin surviving fractions were high, around 10% in N-free stress medium (HAS-N), but was around five orders of magnitude lower in complete medium (HAS-Comp; Figure 2A and Supplementary Figure 5A). In contrast, only the cultures pregrown to Nlim stationary phase had high surviving fractions close to 10% in HAS-N medium with ciprofloxacin and streptomycin. The surviving fractions of exponential and Clim cells in HAS-N were 2–4 orders of magnitude lower and were independent of the HAS medium (Figures 2B,C; Supplementary Figures 5B,C).

In summary, antibiotic survival varies with both modes of action of the antibiotic, the pregrown physiological state, and the stress medium. The overall trend is that if the cells sense complete growth-supporting conditions, they continue or reinitiate growth. Active growth makes the cells much more sensitive to antibiotics, with the fatal consequence that the surviving fractions drastically decrease. On the other hand, with conditions not facilitating growth, the surviving fractions were as high as up to > 10%. Therefore, careful experimental design focusing on one-factor variation is an absolute necessity for obtaining results that can be unambiguously interpreted.

### 3.3. *Escherichia coli* Δ*relA* cells are less prepared for survival in N-limited conditions

We explored both carbon-and nitrogen-limited stationary phase cultures in the present study. Since the growth limitations differ, it will evoke different stress signaling mechanisms for the cell to adapt to the new environment and eventually progress into a dormant state. The former has an exogenous nitrogen source but lacks carbon and energy source. In contrast, the latter has a supply of carbon and energy sources but can only run new protein synthesis by turnover of internal nitrogen sources, making long-term survival different in the two states.

In this context, it was interesting to study HAS-survival in an *E. coli* Δ*relA* mutant impaired in its nutrient stress response (Chau et al., 2021; Svenningsen et al., 2021). (p)ppGpp synthesis is shown to be considerably reduced in the Δ*relA* mutant in both Nlim and Clim compared to the WT (Brown, 2019; Svenningsen et al., 2021). The Δ*relA* mutant had the same growth rate in the exponential phase as the WT, but the lag phase was slightly longer (see representative growth curves in Supplementary Figure 4). The exponential and Clim-grown Δ*relA* cells had similar but slightly lower survival than WT for most conditions (Figure 3, black and dotted bars, respectively. WT results from Figure 2 are included to ease comparison between the two strains. Individual values are presented in Supplementary Figure 6). The same trend was observed for most Nlim-grown cells, except for treatment with ciprofloxacin or streptomycin in N-free media. In these conditions, the surviving fractions of the Δ*relA* mutant were two orders of magnitude lower than the WT cells (Figures 3B,F, white bars). This indicates that the deletion of *relA* makes the cell slightly more vulnerable to antibiotic treatment in some conditions and considerably more vulnerable to ciprofloxacin in N-limited conditions. The increased vulnerability of the Δ*relA* cells was not unexpected due to the known role of RelA-dependent synthesis of (p)ppGpp during the stringent response and amino acid starvation (Chau et al., 2021). These results are in line with other studies on survival after high doses of ampicillin, gentamicin, or norfloxacin, even though the assay conditions differ from the ones used here (Hansen et al., 2008; Kudrin et al., 2017; Liu et al., 2017; Sinha et al., 2019; Svenningsen et al., 2019).

### 3.4. Variation of the metabolic state in different pregrown states and different stress media

The metabolic state of any biological cell defines its ability to perform work, i.e., continue growing, sustain maintenance processes, and handle exogenous and endogenous stress. Central metabolism is at the core of all these processes, providing energy and carbon precursors. The composition of the central metabolite pools is the closest snapshot to defining the metabolic state. The potential role of the important energy metabolite ATP availability in persister cell formation has previously been explored (Braetz et al., 2017; Shan et al., 2017; Manuse et al., 2021). Thus, searching for any correlation between the metabolic state and antibiotic efficacy was interesting.

*Escherichia coli* WT and *ΔrelA* mutant cells in exponential and Nlim stationary phases were sampled for metabolite profiling. Three quantitative MS-based methods were applied to absolutely quantify almost 60 central carbon metabolites. The absolute concentrations from the metabolite profiling are presented in Supplementary Table 1, and metabolite abbreviations and descriptions are presented in Supplementary Table 2. As expected, exponentially grown *E. coli* WT and *ΔrelA* cultures clustered together in the PCA scores plot since RelA does not have an active role during unlimited growth conditions (Figure 4A; Chau et al., 2021). On the other hand, there was a distinct separation along PC1 with the *ΔrelA* most distant from the exponential cells for the stationary grown cultures. Inspection of individual metabolite levels revealed significant changes from exponential to Nlim stationary phase for 16 and 22 metabolites for the WT and *ΔrelA* mutant, respectively (*T*-test with FDR-adjusted value of *p* < 0.05, with log2 fold change threshold at 1). In WT, most metabolites in the tricarboxylic acid (TCA) cycle accumulated, while upper glycolytic and pentose phosphate pathway (PPP) metabolite pools decreased when entering Nlim stationary phase. In the nucleoside phosphate pools, there were no changes in the AXP pools (the ATP, ADP, and AMP series with X for the number of phosphate groups) slight decrease in the GXP pools, while the UXP pools increased (Figure 4B). Many amino acids accumulated, probably as a consequence of the shutdown of new protein synthesis. The substantial accumulation of the important regulatory metabolite α-ketoglutarate (aKG) is expected according to the literature on response to nitrogen limitation (Chubukov et al., 2014; Huergo and Dixon, 2015). The same overall picture of the adaptation to Nlim is obtained in the *ΔrelA* mutant. However, the changes are stronger, as indicated by larger separation from the exponential phase samples along PC1 (Figure 4A) and by larger log2-fold changes than the WT (Figure 4B).

While the *E. coli* WT maintained the same ATP level when entering the stationary phase, a significant reduction of 40% was observed in the *ΔrelA* mutant (Figure 4C). The adenylate energy charge (AEC) is the ratio of ATP, ADP, and AMP, which express the energetic status of a cell (Atkinson and Walton, 1967). This was slightly lowered in the mutant but not to the same degree as the absolute levels of ATP (Figure 4D). Comparing the stationary phase-grown *ΔrelA* versus WT revealed that most glycolytic and TCA cycle metabolite pools were higher while most amino acids were lower in *ΔrelA* (Supplementary Figure 7). Upon starvation, (p)ppGpp inhibits degradation of polyphosphate, which leads to an accumulation of free amino acids (Dalebroux and Swanson, 2012), which we observed to a higher degree in the *ΔrelA* mutant than in the WT. The most pronounced difference between the *ΔrelA* mutant and the WT was the 75 times higher phosphoribosyl pyrophosphate (PRPP) level in the mutant. The larger effect on the metabolite pools of the *ΔrelA* mutant indicates weaker homeostatic mechanisms in the absence of the main (p)ppGpp synthesizing step during nitrogen starvation. This further strengthens the emerging role of (p)ppGpp in stress regulation beyond the regulation of transcription, as it directly affects the central metabolism (Chau et al., 2021).

**Figure 5:**
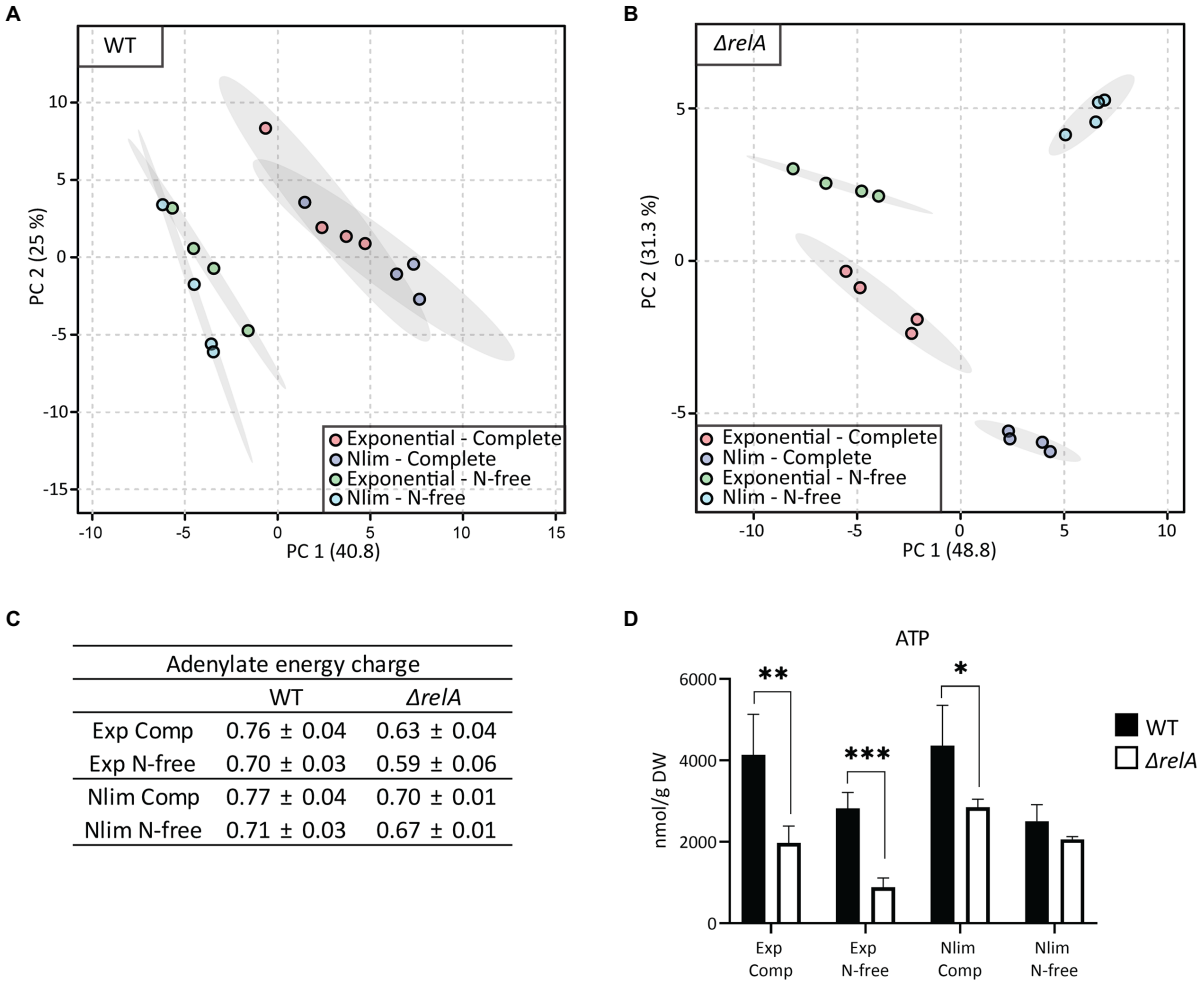
Metabolite profiling of *E. coli* WT and *ΔrelA* from two pregrown states resuspended into complete or N-free media. Aliquots from either exponential phase or nitrogen-limited stationary phase were resuspended in either complete or N-free media, and samples were taken for metabolic analysis immediately after resuspension. Principal component analysis (PCA) with 57 metabolites was performed for **(A)** WT and **(B)** *ΔrelA* samples. **(C)** Adenylate energy charge (AEC) was calculated using the absolute concentrations of ATP, ADP, and AMP, with *n* = 4. **(D)** Absolute ATP concentrations, with unpaired, two-tailed t-test between each condition, *n* = 4. ^*^p ≤ 0.05, ^**^p ≤ 0.01, ^***^p ≤ 0.001. Comp, Complete HAS-medium; N-free, HAS medium lacking nitrogen; Exp, exponential phase; Nlim, nitrogen-limited stationary phase.

The HAS assay includes a centrifugation step before resuspension into stress media with antibiotics, and our next aim was to explore how this affects the metabolic state of the cells. Thus, metabolite profiling was also performed immediately after resuspension into complete or N-free media (this time without antibiotics). Even though the metabolite sampling was done as fast as possible after resuspension, the WT samples clustered based on the stress media and not on the pregrown cultivation state (Figure 5A). Surprisingly, the cells immediately lost their pregrown metabolic state and adapted their metabolite pool levels to the new conditions. The response was clearly not only due to the centrifugation step itself, as we see two different clusters based on the stress media (that is, N-free and complete media samples are separated). The *ΔrelA* cells were slower in their response, as indicated by the clear separation along both PC1 and PC2, with separation based on pregrown state and resuspension media, respectively (Figure 5B). Especially exponentially grown *ΔrelA* cells are less able to respond to changed incubation conditions, since a significant decrease in absolute ATP concentration and AEC was observed for both complete and N-free resuspension (Figures 5C,D).

In summary, both HAS experiments and metabolite profiling highly depend on the stress media. Very low surviving fractions are obtained in conditions supporting growth (complete medium), independent of the antibiotic mode of action. If the HAS stress medium is growth deficient (N-free medium), the surviving fractions depend on the antibiotic mode of action and pregrown cultivation state. Both HAS and metabolite profiling data show that the *ΔrelA* mutant is less fit for survival, especially in N-free conditions.

## 4. Discussion

Experimentation on antibiotic tolerance and survival, and stress response studies in general, are challenging to design due to multiple variables, such as pregrown state, medium composition during both growth and stress phases, and type and dose of antibiotics. This report demonstrates the importance of designing one-variable assays in high-dose antibiotics studies. We studied the persister assay with *E. coli* and found the value of including two different media during HAS. Survival differed for the different antibiotics, stress media, and pregrown states. The combination of Nlim stationary-grown cells in N-free stress medium supports the highest surviving fraction, around 10%, for all three antibiotics. The mechanism behind the high survival in this condition is likely not the same for all three antibiotics. Energy dependency for uptake of the antibiotics differs and cannot be a universal explanation for the increased survival in N-free conditions since streptomycin requires the proton-motive force for entry to bacteria (Krause et al., 2016), but ampicillin and ciprofloxacin do not (Vergalli et al., 2019). Ampicillin exhibits growth-rate dependent lysis, with a very low killing in non-growing states (Eng et al., 1991; Lee et al., 2018). This is due to the inhibition of cell wall synthesis this antibiotic imposes, and the high survival in all HAS with N-free media is as expected. Ciprofloxacin, on the other hand, is known to kill both growing and non-growing bacteria (Sanders, 1988), which is not in line with the relatively high survival after the combination of Nlim in N-free conditions (Figures 2B, 3B).

In Harms et al. (2017), reported on the preference to use defined mineral medium instead of complex rich medium, such as LB, for the study of HAS, which we also employ in our study. The choice of medium is critical, but the choice of cultivation vessel is also very important. Bioreactors are preferred as they provide total control of the culture, with constant pH and oxygenation level. Also, sampling can be done directly from the vessel without disturbing the cells, which is done when shake flasks are transferred from a shaking cabinet. Harms and co-workers used the most common version of the persister assay by adding antibiotics directly into the growth media at various time points and hence different growth stages. This is comparable to our HAS complete condition for most exponential phase samples (although without the centrifugation step). They reported increasing survival with increasing culture age, from exponential into stationary growth phase for all tested antibiotics (gentamycin, ampicillin, and ciprofloxacin). In this setup, the culture media background varies at the different sampling points, and the reason for entry to stationary phase is not defined (a limiting nutrient can be controlled in a defined medium; Harms et al., 2017). In our setup, on the other hand, we show that survival increases just because of a more nutrient-poor condition. Gutierrez et al. (2017) studied ciprofloxacin survival at different bacterial densities. They found that the starvation process, rather than cellular adaptation to starvation, is the driving force when high-density cultures become more tolerant to ciprofloxacin than low-density cultures. The bacteria become more sensitive when they supplement the cultures with both a carbon source and oxygen (Gutierrez et al., 2017). However, in our settings, we have the same bacterial density and oxygen availability in all HAS conditions and find that the availability of nitrogen also impacts the ciprofloxacin sensitivity (Figure 2B). Gutierrez *et al.* also pointed to the importance of experimental conditions, including growth conditions, when studying antibiotic sensitivity, which our study also emphasizes.

The variability in survival between some of the experiments presented in Figures 1–3 is relatively high, especially for streptomycin. In general, the variation can partly be explained by very low numbers since small differences in survival will have large effects on survival fractions. However, others have also reported higher variation for aminoglycoside antibiotics than for ciprofloxacin and ampicillin (Harms et al., 2017). It has been reported that the persister survival after ciprofloxacin and streptomycin treatment correlates with the concentration, while the survival after, e.g., ampicillin treatment does not (Abel Zur Wiesch et al., 2015). The ciprofloxacin concentration used herein was 500 × the MIC, while the streptomycin concentration was only 20 × the MIC. This can partly explain the higher variation between the biological replicates for streptomycin, given that small variations in bacterial density or antibiotic concentrations could influence survival, while the concentration of ciprofloxacin is high enough to avoid this possibility.

Brown studied ciprofloxacin-survival of exponential and N-limited *E. coli* cells and used centrifugation of the pregrown cells before antibiotic treatment, but limited the study to what corresponds to our complete stress medium (Brown, 2019). His results align with ours for the *E. coli* WT and *ΔrelA* mutant 4 h after treatment, with an approximately one order of magnitude higher survival for Nlim than the exponential phase (Figure 3A, open and black bars. Note that our results are from 5 h after treatment). However, Brown also reported survival after 24 h of treatment, where the same trend is seen for the WT. For the *ΔrelA* mutant, on the other hand, there was clearly lower survival for the Nlim cells compared with the exponential phase after 24 h (Brown, 2019). This goes in the same direction as our results: the *ΔrelA* mutant is less fit for survival in Nlim than the WT.

High-antibiotic stress in growth-limiting conditions (Figures 3B,D,F, and initial N-/C-/CN-free conditions in Figure 1) provides complementary and important information to the HAS complete data. Bacterial cells are nutrient-limited in many situations where they are exposed to exogenous stress. This includes clinical situations in biofilms, where nutritional access is restricted. *E. coli* isolated from urinary tract infections exhibit gene expression profiles that are representative of nitrogen limitation, both in bacteria isolated from humans and mice (Snyder et al., 2004; Hagan et al., 2010; Switzer et al., 2018). Nitrogen limitation is thus a clinically relevant situation, which makes it highly relevant to include HAS in growth-limiting conditions in studies on antibiotic exposure and measurements of surviving fractions. The surviving fractions were high in HAS N-free conditions after Nlim stationary phase (> 1–10%). Growth-supporting supernatant from exponentially growing cultures cannot be diluted into a HAS N-free environment without a medium-removal step. This could be centrifugation, as in this study, or filtration, the latter used by Svenningsen et al. (2021). Future improvements to the persister assay could be centrifugation-independent systems for faster media exchange, such as cross-flow filtration systems (Raub et al., 2015; Busche et al., 2019). The importance of assay incubation conditions is also displayed by the metabolite profiling results, where *E. coli* WT cells respond very fast to changes in incubation conditions (Figure 5A). The speed of this was surprising and is important knowledge for future protocol establishments and research.

ATP pools have been investigated for their potential roles in persister cell formation (Braetz et al., 2017; Shan et al., 2017). Shan and co-workers concluded that stochastic variation in ATP levels is the primary mechanism of persister formation and reported lower ATP levels in stationary grown than exponential phase *E. coli* cultures (Shan et al., 2017). Contrastingly, we observed similar ATP levels in exponential and Nlim stationary-grown *E. coli* WT cultures. Differences in nutrient depletion in the stationary phase or time since entry into the stationary phase might explain these apparently conflicting results (details on the timing of experiments are not provided in Shan et al. (2017). Different assays to measure ATP levels were also used. The MS-based metabolite profiling used in our study also enables the calculation of the AEC since both AMP, ADP, and ATP are quantified. The AEC is an as interesting and physiologically relevant indicator as ATP levels alone (Lu et al., 2017). We found that the AEC did not drop in Nlim stationary phase *E. coli* WT cultures or the exponential phase in our experimental setup (Figure 4D). Additionally, we also find spontaneous persistence occurring at very low frequencies in our assays. Hence, global population assays such as MS-based metabolite profiling (used in our study) or ATP bioluminescence kit [used by Shan et al. (2017)] cannot determine if stochastic variations in ATP levels cause this persistence. Entry into the stationary phase leads to the induction of nutritionally triggered survival mechanisms. The high surviving fractions (around 10%) cannot be linked to stochastic variation in ATP levels alone but rather to how the cells prepare for long-term survival by well-regulated mechanisms. Global population-based metabolite profiling could detect trends in such large sub-populations, but our study did not observe this (Figure 4).

Interestingly, the only large difference in surviving fractions between the *E. coli* WT and the Δ*relA* mutant was for Nlim stationary grown cells incubated in HAS N-free conditions with ciprofloxacin and streptomycin (Figures 3B,F, white bars). This condition corresponds to the induction of triggered persisters, and the *E. coli* WT has a higher level of these survivors than the Δ*relA* mutant. The metabolite profiling data indicates that the Δ*relA* mutant responded slower to altered environmental conditions than the *E. coli* WT (Figures 5A,B), which indicates an impaired stress response in this mutant. The very fast response in the WT further supports the notion that (p)ppGpp has roles in the cell beyond transcriptional regulation (Dalebroux and Swanson, 2012; Hauryliuk et al., 2015; Chau et al., 2021). It has also recently been suggested that reduced (p)ppGpp levels result in reduced steady-state expression of metabolic enzymes, which also might explain the reduced response at the metabolome level (Zhu and Dai, 2019). Almost no difference between the survival of exponential phase *E. coli* WT and Δ*relA* mutant cells (Figure 3) indicates that (p)ppGpp is not important in the formation of spontaneous persisters.

On the single metabolite level, the 70 times higher phosphoribosyl pyrophosphate (PRPP) level in Nlim stationary grown Δ*relA* mutant cells compared to the *E. coli* WT is most noticeable (Supplementary Figure 7). PRPP is an important precursor metabolite that links the pentose phosphate pathway (PPP) to nucleotide biosynthesis, and control of PRPP level has recently been suggested to be controlled by a (p)ppGpp-regulated enzyme (Wang et al., 2020). However, among the nucleoside phosphates, there is no common response (AXPs and CXPs decrease, while GXPs and UXPs increase). The requirement for nucleoside phosphates in cells decreases when growth ceases. Hence, the large accumulation of PRPP in the Δ*relA* mutant indicates that these cells are less able to respond fast enough to balance nucleotide synthesis versus consumption. Other metabolites of the central metabolism, namely aKG, glutamate (Glu), and glutamine (Gln), which are important in the control of nitrogen metabolism, are similar in the *E. coli* WT and the Δ*relA* mutant. These are interesting observations for future studies on the role of (p)ppGpp in stress response and metabolic regulation.

Here we have studied a modified version of the persister assay, showing the importance of a one-variable study design to obtain data for easier interpretation and comparisons between the experimental variables. The aim of a study must guide the study design in future projects and the choice of protocols as both under-and overestimation of survival may happen with the wrong choice.

## Data availability statement

The original contributions presented in the study are included in the Supplementary material, further inquiries can be directed to the corresponding author.

## Author contributions

LT and PB: conceptualization, methodology, funding acquisition, and writing–original draft preparation. LT: formal analysis and visualization. LT (lead), GB, and JB: investigation. LT, GB, JB, and PB: writing–review and editing. All authors contributed to the article and approved the submitted version.

## Funding

This research was funded by the Trond Mohn foundation (TAMiR project: TMS2019TMT05) and by internal funding from The Department of Biotechnology and Food Science, Norwegian University of Science and Technology (NTNU). The open access publication fee was funded by NTNU.

## Conflict of interest

The authors declare that the research was conducted in the absence of any commercial or financial relationships that could be construed as a potential conflict of interest.

## Publisher’s note

All claims expressed in this article are solely those of the authors and do not necessarily represent those of their affiliated organizations, or those of the publisher, the editors and the reviewers. Any product that may be evaluated in this article, or claim that may be made by its manufacturer, is not guaranteed or endorsed by the publisher.

## References

[ref1] Abel Zur Wiesch P. Abel S. Gkotzis S. Ocampo P. Engelstädter J. Hinkley T.. (2015). Classic reaction kinetics can explain complex patterns of antibiotic action. Sci. Transl. Med. 7:287ra73. doi: 10.1126/scitranslmed.aaa8760, PMID: 25972005 PMC4554720

[ref2] Andrews J. M. (2001). Determination of minimum inhibitory concentrations. J. Antimicrob. Chemother. 48, 5–16. doi: 10.1093/jac/48.suppl_1.5 11420333

[ref3] Atkinson D. E. Walton G. M. (1967). Adenosine triphosphate conservation in metabolic regulation: rat liver citrate cleavage enzyme. J. Biol. Chem. 242, 3239–3241. doi: 10.1016/S0021-9258(18)95956-9 6027798

[ref4] Baba T. Ara T. Hasegawa M. Takai Y. Okumura Y. Baba M.. (2006). Construction of Escherichia coli K-12 in-frame, single-gene knockout mutants: the Keio collection. Mol. Syst. Biol. 2, 1–11. doi: 10.1038/msb4100050 PMC1681482 16738554

[ref5] Balaban N. Q. Helaine S. Lewis K. Ackermann M. Aldridge B. Andersson D. I.. (2019). Definitions and guidelines for research on antibiotic persistence. Nat. Rev. Microbiol. 17, 441–448. doi: 10.1038/s41579-019-0196-3 30980069 PMC7136161

[ref6] Bigger J. W. (1944). Treatment of staphylococcal infections with penicillin. Lancet 244, 497–500. doi: 10.1016/s0140-6736(00)74210-3

[ref7] Braetz S. Schwerk P. Thompson A. Tedin K. Fulde M. (2017). The role of ATP pools in persister cell formation in (fluoro)quinolone-susceptible and-resistant strains of *Salmonella enterica* ser *typhimurium*. Vet. Microbiol. 210, 116–123. doi: 10.1016/j.vetmic.2017.09.007 29103680

[ref8] Brown D. R. (2019). Nitrogen starvation induces Persister cell formation in *Escherichia coli*. J. Bacteriol. 201, e00622–e00618. doi: 10.1128/jb.00622-18 30420451 PMC6349093

[ref9] Busche J. F. Möller S. Stehr M. Dietzel A. (2019). Cross-flow filtration of *Escherichia coli* at a nanofluidic gap for fast immobilization and antibiotic susceptibility testing. Micromachines 10:691. doi: 10.3390/mi10100691, PMID: 31614761 PMC6843207

[ref10] Chau N. Y. E. Ahmad S. Whitney J. C. Coombes B. K. (2021). Emerging and divergent roles of pyrophosphorylated nucleotides in bacterial physiology and pathogenesis. PLoS Pathog. 17:e1009532. doi: 10.1371/journal.ppat.1009532 33984072 PMC8118318

[ref11] Chong J. Wishart D. S. Xia J. (2019). Using MetaboAnalyst 4.0 for comprehensive and integrative metabolomics data analysis. Curr. Protoc. Bioinform. 68:e86. doi: 10.1002/cpbi.86 31756036

[ref12] Chowdhury N. Kwan B. W. Wood T. K. (2016). Persistence increases in the absence of the Alarmone guanosine Tetraphosphate by reducing cell growth. Sci. Rep. 6, 1–9. doi: 10.1038/srep20519, PMID: 26837570 PMC4738310

[ref13] Chubukov V. Gerosa L. Kochanowski K. Sauer U. (2014). Coordination of microbial metabolism. Nat. Rev. Microbiol. 12, 327–340. doi: 10.1038/nrmicro3238 24658329

[ref14] Dalebroux Z. D. Swanson M. S. (2012). ppGpp: magic beyond RNA polymerase. Nat. Rev. Microbiol. 103, 203–212. doi: 10.1038/nrmicro2720 PMC13198741 22337166

[ref15] Datsenko K. A. Wanner B. L. (2000). One-step inactivation of chromosomal genes in Escherichia coli K-12 using PCR products. Proc. Natl. Acad. Sci. U. S. A. 97, 6640–6645. doi: 10.1073/pnas.120163297 10829079 PMC18686

[ref16] Dörr T. Vulić M. Lewis K. (2010). Ciprofloxacin causes Persister formation by inducing the TisB toxin in *Escherichia coli*. PLoS Biol. 8:e1000317. doi: 10.1371/JOURNAL.PBIO.1000317 20186264 PMC2826370

[ref17] Droste P. Nöh K. Wiechert W. (2013). Omix–a visualization tool for metabolic networks with highest usability and customizability in focus. Chemie Ing. Tech. 85, 849–862. doi: 10.1002/cite.201200234

[ref18] Eng R. H. K. Padberg F. T. Smith S. M. Tan E. N. Cherubin C. E. (1991). Bactericidal effects of antibiotics on slowly growing and nongrowing bacteria. Antimicrob. Agents Chemother. 35, 1824–1828. doi: 10.1128/aac.35.9.1824 1952852 PMC245275

[ref19] Goormaghtigh F. Fraikin N. Putrins M. Hallaert T. Hauryliuk V. Garcia-Pino A.. (2018). Reassessing the role of type II toxin-antitoxin systems in *Escherichia coli* type II Persister cells. MBio 9, 1–14. doi: 10.1128/mBio.00640-18, PMID: 29895634 PMC6016239

[ref20] Goormaghtigh F. Van Melderen L. (2016). Optimized method for measuring persistence in *Escherichia coli* with improved reproducibility. Methods Mol. Biol. 1333, 43–52. doi: 10.1007/978-1-4939-2854-5_4 26468098

[ref21] Gutierrez A. Jain S. Bhargava P. Hamblin M. Lobritz M. A. Collins J. J. (2017). Understanding and sensitizing density-dependent persistence to quinolone antibiotics. Mol. Cell 68, 1147–1154.e3. doi: 10.1016/j.molcel.2017.11.012 29225037

[ref22] Hagan E. C. Lloyd A. L. Rasko D. A. Faerber G. J. Mobley H. L. T. (2010). Escherichia coli global gene expression in urine from women with urinary tract infection. PLoS Pathog. 6:e1001187. doi: 10.1371/journal.ppat.1001187 21085611 PMC2978726

[ref23] Hansen S. Lewis K. Vulić M. (2008). Role of global regulators and nucleotide metabolism in antibiotic tolerance in Escherichia coli. Antimicrob. Agents Chemother. 52, 2718–2726. doi: 10.1128/aac.00144-08, PMID: 18519731 PMC2493092

[ref24] Harms A. Fino C. Sørensen M. A. Semsey S. Gerdes K. (2017). Prophages and growth dynamics confound experimental results with antibiotic-tolerant Persister cells. MBio 8, 1–18. doi: 10.1128/mBio.01964-17, PMID: 29233898 PMC5727415

[ref25] Hauryliuk V. Atkinson G. C. Murakami K. S. Tenson T. Gerdes K. (2015). Recent functional insights into the role of (p)ppGpp in bacterial physiology. Nat. Rev. Microbiol. 13, 298–309. doi: 10.1038/nrmicro3448 25853779 PMC4659695

[ref26] Hengge R. (2011). “The general stress response in Gram-negative bacteria” in Bacterial Stress Responses. eds. Storz G. Hengge R. (Washington, DC: ASM Press), –251, 289.

[ref27] Hobby G. L. Meyer K. Chaffee E. (1942). Observations on the mechanism of action of penicillin. Exp. Biol. Med. 50, 281–285. doi: 10.3181/00379727-50-13773

[ref28] Holden D. W. Errington J. (2018). Type II toxin-antitoxin systems and persister cells. MBio 9, 10–11. doi: 10.1128/mBio.01574-18, PMID: 30254124 PMC6156201

[ref29] Huergo L. F. Dixon R. (2015). The emergence of 2-Oxoglutarate as a master regulator metabolite. Microbiol. Mol. Biol. Rev. 79, 419–435. doi: 10.1128/MMBR.00038-15 26424716 PMC4651028

[ref30] Irazoki O. Hernandez S. B. Cava F. (2019). Peptidoglycan muropeptides: release, perception, and functions as signaling molecules. Front. Microbiol. 10, 1–17. doi: 10.3389/fmicb.2019.00500 30984120 PMC6448482

[ref31] Kim J. S. Wood T. K. (2016). Persistent persister misperceptions. Front. Microbiol. 07, 1–7. doi: 10.3389/fmicb.2016.02134, PMID: 28082974 PMC5187198

[ref32] Kim J. Wood T. K. (2017). Tolerant, growing cells from nutrient shifts are not Persister cells. MBio 8, 1–5. doi: 10.1128/mBio.00354-17 PMC5395667 28420737

[ref33] Kim J. S. Yamasaki R. Song S. Zhang W. Wood T. K. (2018). Single cell observations show persister cells wake based on ribosome content. Environ. Microbiol. 20, 2085–2098. doi: 10.1111/1462-2920.14093 29528544

[ref34] Krause K. M. Serio A. W. Kane T. R. Connolly L. E. (2016). Aminoglycosides: an overview. Cold Spring Harb. Perspect. Med. 6, 1–18. doi: 10.1101/cshperspect.a027029, PMID: 27252397 PMC4888811

[ref35] Kudrin P. Varik V. Raquel S. Oliveira A. Beljantseva J. Del T.. (2017). Subinhibitory concentrations of bacteriostatic antibiotics induce relA-dependent and relA-independent tolerance to β-lactams. Antimicrob. Agents Chemother. 61, 1–17. doi: 10.1128/aac.02173-16, PMID: 28115345 PMC5365698

[ref36] Kvitvang H. F. N. Kristiansen K. A. Bruheim P. (2014). Assessment of capillary anion exchange ion chromatography tandem mass spectrometry for the quantitative profiling of the phosphometabolome and organic acids in biological extracts. J. Chromatogr. A 1370, 70–79. doi: 10.1016/j.chroma.2014.10.029 25454131

[ref37] Lee A. J. Wang S. Meredith H. R. Zhuang B. Dai Z. You L. (2018). Robust, linear correlations between growth rates and β-lactam–mediated lysis rates. Proc. Natl. Acad. Sci. 115, 4069–4074. doi: 10.1073/pnas.1719504115, PMID: 29610312 PMC5910845

[ref38] Levin-Reisman I. Ronin I. Gefen O. Braniss I. Shoresh N. Balaban N. Q. (2017). Antibiotic tolerance facilitates the evolution of resistance. Science 355, 826–830. doi: 10.1126/science.aaj2191 28183996

[ref39] Lewis K. (2020). The science of antibiotic discovery. Cells 181, 29–45. doi: 10.1016/j.cell.2020.02.056 32197064

[ref40] Liu S. Wu N. Zhang S. Yuan Y. Zhang W. (2017). Variable Persister gene interactions with (p)ppGpp for Persister formation in *Escherichia coli*. Front. Microbiol. 8, 1–14. doi: 10.3389/fmicb.2017.01795, PMID: 28979246 PMC5611423

[ref41] Lu W. Su X. Klein M. S. Lewis I. A. Fiehn O. Rabinowitz J. D. (2017). Metabolite measurement: pitfalls to avoid and practices to follow. Annu. Rev. Biochem. 86, 277–304. doi: 10.1146/annurev-biochem-061516-044952 28654323 PMC5734093

[ref42] Manuse S. Shan Y. Canas-Duarte S. J. Bakshi S. Sun W. S. Mori H.. (2021). Bacterial persisters are a stochastically formed subpopulation of low-energy cells. PLoS Biol. 19:e3001194. doi: 10.1371/journal.pbio.3001194, PMID: 33872303 PMC8084331

[ref43] Meylan S. Andrews I. W. Collins J. J. (2018). Targeting antibiotic tolerance, pathogen by pathogen. Cells 172, 1228–1238. doi: 10.1016/j.cell.2018.01.037 29522744

[ref44] Murray C. J. Ikuta K. S. Sharara F. Swetschinski L. Robles Aguilar G. Gray A.. (2022). Global burden of bacterial antimicrobial resistance in 2019: a systematic analysis. Lancet 399, 629–655. doi: 10.1016/s0140-6736(21)02724-0, PMID: 35065702 PMC8841637

[ref45] Pacios O. Blasco L. Bleriot I. Fernandez-Garcia L. Ambroa A. López M.. (2020). (p)ppGpp and its role in bacterial persistence: new challenges. Antimicrob. Agents Chemother. 64, e01283–e01220. doi: 10.1128/AAC.01283-20 32718971 PMC7508602

[ref46] Raub C. B. Lee C. Kartalov E. (2015). Sequestration of bacteria from whole blood by optimized microfluidic cross-flow filtration for rapid antimicrobial susceptibility testing. Sensors Actuators B Chem. 210, 120–123. doi: 10.1016/j.snb.2014.10.061

[ref47] Røst L. M. Thorfinnsdottir L. B. Kumar K. Fuchino K. Langørgen I. E. Bartosova Z.. (2020). Absolute quantification of the central carbon metabolome in eight commonly applied prokaryotic and eukaryotic model systems. Meta 10, 1–7. doi: 10.3390/metabo10020074, PMID: 32093075 PMC7073941

[ref48] Sanders C. C. (1988). Ciprofloxacin: in vitro activity, mechanism of action, and resistance. Rev. Infect. Dis. 10, 516–527. doi: 10.1093/clinids/10.3.516 3293157

[ref49] Shan Y. Brown Gandt A. Rowe S. E. Deisinger J. P. Conlon B. P. Lewis K. (2017). ATP-dependent persister formation in *Escherichia coli*. MBio 8, 1–14. doi: 10.1128/mBio.02267-16 PMC5296605 28174313

[ref50] Sinha A. K. Winther K. S. Roghanian M. Gerdes K. (2019). Fatty acid starvation activates RelA by depleting lysine precursor pyruvate. Mol. Microbiol. 112, 1339–1349. doi: 10.1111/mmi.14366 31400173

[ref51] Snyder J. A. Haugen B. J. Buckles E. L. Lockatell C. V. Johnson D. E. Donnenberg M. S.. (2004). Transcriptome of uropathogenic Escherichia coli during urinary tract infection. Infect. Immun. 72, 6373–6381. doi: 10.1128/iai.72.11.6373-6381.2004 15501767 PMC523057

[ref52] Stafsnes M. H. Røst L. M. Bruheim P. (2018). Improved phosphometabolome profiling applying isotope dilution strategy and capillary ion chromatography-tandem mass spectrometry. J. Chromatogr. B 1083, 278–283. doi: 10.1016/j.jchromb.2018.02.004 29571119

[ref53] Svenningsen M. S. Svenningsen S. L. Sørensen M. A. Mitarai N. (2021). Existence of log-phase *Escherichia coli* persisters and lasting memory of a starvation pulse. Life Sci. Alliance 5:e202101076. doi: 10.26508/lsa.202101076 34795016 PMC8605324

[ref54] Svenningsen M. S. Veress A. Harms A. Mitarai N. Semsey S. (2019). Birth and resuscitation of (p)ppGpp induced antibiotic tolerant Persister cells. Sci. Rep. 9:6056. doi: 10.1038/s41598-019-42403-7 30988388 PMC6465370

[ref55] Switzer A. Brown D. R. Wigneshweraraj S. (2018). New insights into the adaptive transcriptional response to nitrogen starvation in Escherichia coli. Biochem. Soc. Trans. 46, 1721–1728. doi: 10.1042/bst20180502 30514772 PMC6299236

[ref56] Thorfinnsdottir L. B. García-Calvo L. Bø G. H. Bruheim P. Røst L. M. (2023). Optimized fast filtration-based sampling and extraction enables precise and absolute quantification of the *Escherichia coli* central carbon metabolome. Meta 13:150. doi: 10.3390/metabo13020150 PMC9965072 36837769

[ref57] Tkachenko A. G. (2018). Stress responses of bacterial cells as mechanism of development of antibiotic tolerance (review). Appl. Biochem. Microbiol. 54, 108–127. doi: 10.1134/s0003683818020114

[ref58] Vergalli J. Bodrenko I. V. Masi M. Moynié L. Acosta-Gutiérrez S. Naismith J. H.. (2019). Porins and small-molecule translocation across the outer membrane of Gram-negative bacteria. Nat. Rev. Microbiol. 18, 164–176. doi: 10.1038/s41579-019-0294-2 31792365

[ref59] Verstraete L. Van den Bergh B. Verstraeten N. Michiels J. (2022). Ecology and evolution of antibiotic persistence. Trends Microbiol. 30, 466–479. doi: 10.1016/j.tim.2021.10.001 34753652

[ref60] Wang B. Grant R. A. Laub M. T. (2020). ppGpp coordinates nucleotide and amino-acid synthesis in *E. coli* during starvation. Mol. Cell 80, 29–42.e10. doi: 10.1016/j.molcel.2020.08.005 32857952 PMC8362273

[ref61] Wilmaerts D. Windels E. M. Verstraeten N. Michiels J. (2019). General mechanisms leading to Persister formation and awakening. Trends Genet. 35, 401–411. doi: 10.1016/j.tig.2019.03.007 31036343

[ref62] Windels E. M. Michiels J. E. Van Den Bergh B. Fauvart M. Michiels J. (2019). Antibiotics: combatting tolerance to stop resistance. MBio 10. doi: 10.1128/mbio.02095-19 PMC6737247 31506315

[ref63] Zhu M. Dai X. (2019). Growth suppression by altered (p)ppGpp levels results from non-optimal resource allocation in *Escherichia coli*. Nucleic Acids Res. 47, 4684–4693. doi: 10.1093/nar/gkz211, PMID: 30916318 PMC6511861

