# Supplementary material for: Survival of Escherichia coli after high-antibiotic stress is dependent on both the pregrown physiological state and incubation conditions
Source: Front Microbiol. 2023 Mar 10;14:1149978. doi: 10.3389/fmicb.2023.1149978 (PMC10036391; doi:10.3389/fmicb.2023.1149978)
Supplement: Supplementary file 1 [file Data_Sheet_1.PDF]

Supplementary table 1: Intracellular metabolite concentrations in *E. coli* : Intracellular concentrations (nmol/g DW) of all metabolites included in the panel for metabolite profiling, listed for all technical replicates. LOQ; limit of quantification. Metabolite abbreviations are listed in Supplementary table 2.

Strain	Growth phase	Resuspension media	Technical replica	Normalized amount (nmol/ g DW)													
				**2-/3-PG**	**6PG**	**ADP**	**aKG**	**Ala**	**AMP**	**Arg**	**Asn**	**Asp**	**ATP**	**CDP**	**Cit**	**CMP**	**CTP**
ΔrelA	Exponential	None (directly from bioreactor)	1	536	87	2072	248	627	1179	36	135	297	5051	148	341	109	196
			2	584	106	2046	305	482	1157	29	109	238	5192	153	322	114	210
			3	585	122	2244	372	449	1317	39	110	248	5058	173	315	86	199
			4	558	142	2501	84	465	1415	51	136	303	5618	174	324	86	210
		Complete media	1	216	30	1056	62	186	935	30	44	87	1598	119	79	158	77
			2	223	33	1111	70	193	871	30	60	91	1842	156	81	200	113
			3	312	46	1302	118	318	778	24	101	122	2569	198	129	199	204
			4	276	39	1375	104	310	989	53	93	146	1872	305	88	282	152
		N-free media	1	128	23	486	593	130	352	10	7	25	676	49	91	75	40
			2	142	32	586	778	163	511	24	10	48	694	94	103	122	68
			3	211	47	804	1063	227	644	68	20	89	1030	186	139	219	128
			4	170	44	788	1041	178	564	77	18	62	1126	167	125	223	158
	Nlim stationary phase	None (directly from bioreactor)	1	564	51	1224	7628	434	1033	68	143	117	3454	50	1470	211	126
			2	660	47	1319	604	339	955	63	104	111	2714	103	1185	60	119
			3	531	41	1273	7814	391	992	81	94	102	2731	77	1429	196	121
			4	493	42	1164	7022	367	991	63	117	79	3004	51	1268	209	107
		Complete media	1	506	90	1290	299	753	827	122	358	509	2818	487	120	315	582
			2	539	93	1352	255	824	849	93	336	601	2631	539	95	334	564
			3	569	102	1439	483	991	841	54	432	601	3120	480	159	296	599
			4	562	111	1368	371	836	794	93	350	653	2823	349	133	221	391
		N-free media	1	684	93	1135	3615	318	754	135	10	74	2080	225	483	252	248
			2	723	113	1103	4399	406	777	163	14	109	2143	275	599	293	314
			3	724	96	1143	4943	423	761	193	17	91	2020	273	609	315	346
			4	619	104	1105	5059	429	722	213	23	97	2002	260	604	318	340
WT	Exponential	None (directly from bioreactor)	1	624	159	1872	408	538	1094	229	126	286	5637	249	404	196	474
			2	473	115	1397	304	490	717	236	139	307	4733	191	301	153	363
		Complete media	1	405	91	1289	173	405	802	54	108	185	2663	175	130	143	176
			2	652	169	1512	341	644	753	79	216	276	4644	166	217	145	289
			3	582	111	1680	738	619	903	63	177	223	4458	157	305	144	254
			4	636	178	1626	480	715	819	120	230	363	4790	138	225	144	262
		N-free media	1	277	105	1125	1798	305	711	276	52	98	2702	270	217	266	404
			2	328	128	1213	2058	425	686	311	90	148	2959	317	258	310	497
			3	460	113	1524	2692	481	1109	323	110	168	2349	530	252	392	426
			4	496	149	1441	1822	810	794	380	226	295	3270	422	293	410	564
	Nlim stationary phase	None (directly from bioreactor)	1	337	77	1595	3358	656	1240	1503	141	126	5098	242	525	419	503
			2	436	120	1979	3327	757	1315	1626	162	144	5475	315	714	475	485
			3	439	76	1968	3962	775	1336	1574	138	127	4867	306	523	440	450
			4	322	63	1520	3236	588	949	1233	116	101	5119	182	468	400	449
		Complete media	1	529	151	1128	268	497	688	170	316	461	3093	200	138	160	340
			2	702	184	1523	446	825	800	108	457	570	4172	174	245	128	303
			3	744	184	1621	943	893	798	96	480	539	5423	149	360	130	313
			4	644	196	1712	774	796	843	83	379	532	4761	156	273	122	237
		N-free media	1	335	111	836	2130	354	556	265	58	112	1975	157	249	205	276
			2	401	140	1104	2625	497	689	400	115	157	2486	252	329	288	408
			3	470	118	1193	2528	719	726	422	215	237	2968	290	356	369	491
			4	484	106	1301	2288	769	813	447	200	301	2592	351	356	389	411

Supplementary table 1: Continued

Strain	Growth phase	Resuspension media	Technical replica	Normalized amount (nmol/ g DW)													
				**Cys**	**F1,6BP**	**F1P**	**F6P**	**Fum**	**G-/M-1P**	**G6P**	**GA6P**	**GAL1P**	**GDP**	**GL3P**	**Gln**	**Glu**	**Gly**
ΔrelA	Exponential	None (directly from bioreactor)	1	27	1898	63	976	309	67	1100	331	16	471	312	659	3573	546
			2	27	2569	60	1044	323	66	1135	346	20	504	238	512	2752	485
			3	32	1493	82	3227	303	83	3283	553	30	591	355	534	2852	463
			4	32	1710	122	1822	301	101	1939	712	31	590	51	712	3102	503
		Complete media	1	9	757	37	239	279	23	266	60	8	255	75	96	545	181
			2	9	760	38	332	281	27	370	78	7	269	93	159	661	187
			3	9	916	40	397	291	33	436	97	11	273	92	235	1261	276
			4	10	776	36	328	291	27	354	104	11	365	83	232	1153	295
		N-free media	1	8	342	24	279	306	31	325	17	8	130	41	2	310	154
			2	10	381	25	321	295	34	370	18	10	136	40	19	591	186
			3	10	536	35	445	324	46	481	31	16	184	53	13	735	292
			4	11	452	29	473	302	43	442	32	14	162	51	18	766	223
	Nlim stationary phase	None (directly from bioreactor)	1	43	1771	12	1660	778	163	1474	6	92	184	99	14	2834	408
			2	72	1060	12	2117	672	102	1977	10	131	292	170	15	4946	331
			3	52	1285	5	1303	638	151	1310	5	80	225	139	18	3382	305
			4	56	1508	9	1422	728	147	1222	4	76	208	87	5	2093	333
		Complete media	1	9	1758	53	376	269	55	446	190	21	216	66	2169	9143	354
			2	7	1858	49	450	280	49	489	146	18	230	85	2325	10165	380
			3	12	1652	50	498	333	66	502	98	20	237	71	2148	12223	522
			4	11	1987	52	701	304	61	640	148	21	263	96	2039	11409	522
		N-free media	1	11	2378	65	1033	537	113	1033	14	45	247	64	30	3819	310
			2	12	2657	75	1275	507	126	1311	23	57	246	84	58	5134	320
			3	15	2645	68	1428	561	140	1371	21	63	230	61	44	4695	306
			4	14	2367	80	1414	466	143	1477	29	58	221	86	49	4312	301
WT	Exponential	None (directly from bioreactor)	1	29	2651	63	1068	263	63	1097	369	22	330	231	758	4609	542
			2	19	2560	52	611	295	56	701	348	18	267	154	769	3963	554
		Complete media	1	8	1471	40	778	271	32	918	139	13	265	206	532	3193	363
			2	9	2561	58	778	339	72	811	210	20	286	94	818	5906	520
			3	10	1853	51	757	363	63	775	176	19	328	109	447	5714	507
			4	11	2989	58	980	308	68	931	241	23	306	118	966	6683	462
		N-free media	1	10	876	43	523	359	62	564	75	25	196	59	159	3463	280
			2	10	1008	39	683	394	68	825	103	26	196	82	302	5216	307
			3	10	1244	44	692	422	79	726	118	26	310	64	347	6067	342
			4	14	1406	46	814	381	83	848	214	32	275	66	1237	9611	387
	Nlim stationary phase	None (directly from bioreactor)	1	36	833	25	698	359	107	617	82	49	179	95	117	5461	372
			2	31	1121	6	630	491	134	546	68	54	190	122	70	5172	397
			3	39	881	5	1174	334	114	828	87	44	234	211	87	5957	367
			4	36	821	5	632	327	104	611	50	48	171	82	50	4068	338
		Complete media	1	9	2286	40	723	302	58	768	163	20	187	96	2343	8833	671
			2	10	2704	42	792	393	71	789	196	18	277	78	2625	15543	1017
			3	10	2903	47	882	417	83	872	243	21	307	75	2090	16935	845
			4	10	2774	49	1081	377	76	1260	235	17	364	151	1818	14297	588
		N-free media	1	9	1269	36	520	331	58	532	73	30	126	53	283	5248	252
			2	9	1388	42	748	381	71	721	135	30	174	64	354	7399	321
			3	12	1168	40	642	342	81	649	169	47	243	51	748	10353	385
			4	13	1125	37	580	368	74	592	154	49	312	68	855	9421	447

Supplementary table 1: Continued

Strain	Growth phase	Resuspension media	Technical replica	Normalized amount (nmol/ g DW)													
				**GMP**	**GTP**	**His**	**Icit**	**Ile**	**IMP**	**ITP**	**Lac**	**Leu**	**Lys**	**M6P**	**Mal**	**Met**	**PEP**
ΔrelA	Exponential	None (directly from bioreactor)	1	83	2095	90	4	16	396	<LOQ	803	98	587	302	702	94	123
			2	77	2207	83	4	18	392	<LOQ	1172	95	509	327	619	79	156
			3	170	2093	83	6	20	477	<LOQ	683	86	512	817	596	87	191
			4	173	2259	88	5	23	487	<LOQ	869	106	537	704	594	93	202
		Complete media	1	127	698	30	<LOQ	19	229	<LOQ	901	82	188	80	192	27	49
			2	122	731	28	<LOQ	14	255	<LOQ	564	68	215	97	197	37	56
			3	79	965	46	<LOQ	15	250	<LOQ	517	97	240	137	270	44	80
			4	118	704	50	<LOQ	22	278	<LOQ	1096	92	261	105	257	53	82
		N-free media	1	78	392	25	<LOQ	13	90	<LOQ	657	53	141	131	257	35	26
			2	76	344	39	<LOQ	16	108	<LOQ	588	60	142	139	239	37	28
			3	92	458	59	<LOQ	32	154	<LOQ	1963	84	195	194	377	48	48
			4	72	461	40	<LOQ	20	135	<LOQ	980	71	168	187	334	43	42
	Nlim stationary phase	None (directly from bioreactor)	1	25	1478	128	39	51	221	<LOQ	638	124	2526	659	3076	62	225
			2	128	1124	104	28	38	296	<LOQ	372	98	2303	858	2548	51	355
			3	36	1212	101	27	34	206	<LOQ	278	112	2405	549	2663	43	237
			4	25	1305	105	32	36	194	<LOQ	184	95	2374	573	2868	61	247
		Complete media	1	61	921	78	<LOQ	12	214	<LOQ	561	49	468	171	362	40	127
			2	69	825	77	<LOQ	12	227	<LOQ	372	51	435	173	466	55	119
			3	64	962	94	<LOQ	13	228	<LOQ	132	43	432	236	822	80	157
			4	64	913	85	<LOQ	20	229	<LOQ	249	70	383	229	656	95	109
		N-free media	1	78	955	70	12	32	222	<LOQ	488	108	1062	493	1716	51	154
			2	79	941	63	13	33	216	<LOQ	629	122	877	603	1677	53	169
			3	62	875	62	14	36	201	<LOQ	266	105	692	647	1831	68	173
			4	61	839	57	15	31	196	<LOQ	286	100	701	672	1781	59	154
WT	Exponential	None (directly from bioreactor)	1	55	2087	85	10	16	293	<LOQ	706	118	545	298	648	98	149
			2	38	1625	87	3	22	260	<LOQ	1682	114	532	195	528	94	122
		Complete media	1	79	966	69	2	16	291	<LOQ	1105	94	322	177	322	56	89
			2	44	1646	99	3	18	283	<LOQ	577	91	415	290	635	83	135
			3	51	1559	100	4	19	301	<LOQ	416	102	400	296	1013	72	123
			4	39	1681	105	3	21	272	<LOQ	823	96	451	327	783	86	107
		N-free media	1	32	977	61	7	26	224	<LOQ	685	108	356	226	666	65	109
			2	29	1041	59	6	33	212	<LOQ	827	137	384	320	802	72	119
			3	48	870	65	3	35	281	<LOQ	805	145	388	309	951	79	153
			4	36	1128	84	5	49	244	<LOQ	890	207	424	337	721	94	170
	Nlim stationary phase	None (directly from bioreactor)	1	19	1123	129	10	168	267	<LOQ	732	206	2336	264	872	130	241
			2	22	917	164	12	237	366	<LOQ	431	269	2700	277	1324	155	296
			3	35	1073	129	9	177	325	<LOQ	275	208	2547	291	866	134	256
			4	18	1100	104	10	133	246	<LOQ	306	156	2159	268	884	102	199
		Complete media	1	43	992	76	<LOQ	12	240	<LOQ	812	56	403	240	395	64	104
			2	42	1376	101	<LOQ	17	278	<LOQ	738	61	531	301	717	82	181
			3	38	1889	97	<LOQ	16	283	<LOQ	366	57	544	342	1013	87	175
			4	47	1752	97	<LOQ	17	319	<LOQ	900	63	546	352	897	75	142
		N-free media	1	20	625	62	6	26	161	<LOQ	672	100	757	239	680	53	108
			2	26	843	91	6	43	185	<LOQ	912	142	885	302	829	62	172
			3	28	1252	127	5	62	214	<LOQ	458	183	846	288	694	77	216
			4	35	1155	139	4	85	245	<LOQ	1590	223	892	249	724	78	226

Supplementary table 1: Continued

Strain	Growth phase	Resuspension media	Technical replica	Phe	Pro	PRPP	Pyr	R5P	S7P	Ser	Suc	Thr	Trp	Tyr	UDP	UDP-GlcNac	UMP
				**Normalized amount (nmol/ g DW)**													
ΔrelA	Exponential	None (directly from bioreactor)	1	83	140	307	489	300	113	365	3793	342	24	172	369	853	2820
			2	77	120	453	386	262	120	370	5292	293	22	145	377	962	3168
			3	68	126	483	469	218	178	401	5206	306	23	139	435	924	3173
			4	74	136	539	601	233	173	371	6774	356	30	164	436	1109	3559
		Complete media	1	41	44	8	989	179	48	161	1717	82	10	82	268	700	3422
			2	42	38	10	885	157	50	141	1596	85	10	101	396	718	3605
			3	64	72	17	723	156	67	215	2230	132	14	164	523	888	4013
			4	54	65	17	861	150	57	304	1548	147	17	141	665	907	3764
		N-free media	1	19	22	1630	222	214	89	78	1864	43	4	25	309	313	2736
			2	25	29	3151	301	197	100	143	1764	60	6	37	450	355	3409
			3	36	54	4524	436	272	134	270	2064	101	9	50	643	495	4685
			4	30	39	3641	350	227	125	193	1888	72	7	44	517	482	4431
	Nlim stationary phase	None (directly from bioreactor)	1	113	344	8876	328	530	210	381	5697	299	7	106	1949	95	16974
			2	87	255	6945	359	338	272	305	6083	221	5	76	3393	77	15555
			3	100	255	5742	368	449	158	299	4567	228	4	94	2343	105	15562
			4	85	283	7683	267	436	186	289	5501	239	5	72	2031	80	16147
		Complete media	1	48	147	200	561	240	102	219	2236	305	27	189	1260	2676	4697
			2	39	142	249	622	238	101	243	2660	299	26	173	1307	2004	3746
			3	62	172	208	766	224	120	288	3242	358	29	227	1079	2070	3970
			4	53	144	490	809	228	105	413	3077	369	32	208	661	1754	4062
		N-free media	1	64	99	8880	506	716	187	223	4373	174	5	91	1549	541	11459
			2	71	88	10746	406	712	210	269	4626	199	6	103	1461	640	12472
			3	70	77	10419	454	769	234	239	4251	220	5	92	1305	559	12932
			4	74	77	11818	356	750	232	239	4135	207	5	93	1282	421	12918
WT	Exponential	None (directly from bioreactor)	1	95	175	549	263	354	134	341	5442	339	30	175	507	1180	4780
			2	78	177	520	299	253	99	421	2866	343	30	164	393	897	3909
		Complete media	1	61	75	116	828	258	81	345	1952	197	18	177	437	653	3729
			2	81	113	351	788	327	141	477	2992	305	27	235	350	1066	4683
			3	86	100	166	864	228	120	434	3028	268	17	200	414	1099	3626
			4	75	111	391	712	291	141	480	2824	341	27	228	299	1076	3882
		N-free media	1	58	55	387	453	195	113	201	3387	189	10	113	644	683	8815
			2	72	62	842	438	201	137	256	3400	227	15	138	619	755	8599
			3	73	61	961	908	216	140	264	3007	225	17	131	1095	793	8541
			4	102	79	1476	667	232	165	321	2871	337	30	198	783	818	9228
	Nlim stationary phase	None (directly from bioreactor)	1	144	194	102	242	135	106	257	5887	1141	19	210	722	485	11104
			2	125	247	80	292	163	122	255	5701	1370	17	222	841	457	12833
			3	152	195	92	309	150	103	255	5159	1158	21	211	966	454	10842
			4	133	164	117	195	121	103	235	5863	954	14	176	671	424	9890
		Complete media	1	67	146	711	654	353	119	620	2961	274	27	202	572	902	4166
			2	91	216	530	868	343	154	694	3596	341	34	248	359	1198	3442
			3	99	216	422	798	324	167	623	3879	369	30	249	286	1257	3479
			4	84	184	358	652	302	153	592	3794	371	31	223	259	984	3200
		N-free media	1	70	53	1363	407	218	109	244	2997	192	15	136	532	480	7490
			2	92	70	2948	287	259	131	306	4134	282	23	183	535	394	10472
			3	130	82	1468	380	202	140	310	3637	366	39	231	648	428	11822
			4	132	107	704	681	187	127	471	3607	382	41	215	741	460	11345

Supplementary table 1: Continued

Strain	Growth phase	Resuspension media	Technical replica	Normalized amount (nmol/ g DW)	
				UTP	Val
ΔrelA	Exponential	None (directly from bioreactor)	1	998	204
			2	1068	179
			3	1035	177
			4	1108	221
		Complete media	1	439	92
			2	629	85
			3	1153	143
			4	803	127
		N-free media	1	562	49
			2	690	57
			3	976	87
			4	1088	63
	Nlim stationary phase	None (directly from bioreactor)	1	11071	146
			2	8187	106
			3	8312	98
			4	9756	105
		Complete media	1	3294	116
			2	2935	118
			3	2920	100
			4	1729	147
		N-free media	1	3708	124
			2	3406	111
			3	3549	112
			4	3524	99
WT	Exponential	None (directly from bioreactor)	1	2042	200
			2	1547	202
		Complete media	1	936	143
			2	1320	158
			3	1428	120
			4	1147	148
		N-free media	1	2152	129
			2	2235	164
			3	1907	153
			4	2300	233
	Nlim stationary phase	None (directly from bioreactor)	1	3209	244
			2	2805	314
			3	2926	248
			4	3379	202
		Complete media	1	2017	104
			2	1327	95
			3	1175	80
			4	893	99
		N-free media	1	1856	105
			2	1821	133
			3	2296	172
			4	1984	207
