# Supplementary material for: Survival of Escherichia coli after high-antibiotic stress is dependent on both the pregrown physiological state and incubation conditions
Source: Front Microbiol. 2023 Mar 10;14:1149978. doi: 10.3389/fmicb.2023.1149978 (PMC10036391; doi:10.3389/fmicb.2023.1149978)
Supplement: Supplementary file 2 [file Data_Sheet_2.PDF]

**Supplementary table 2.** Metabolite abbreviations. Metabolites included in panel for metabolite profiling, sorted on metabolite class and listed with abbreviation and CAS number. TCA; tricarboxylic acid, PPP; pentose phosphate pathway.

| Class | Abbreviation | Metabolite | CAS number |
|---|---|---|---|
| Glycolysis | G6P | Glucose 6-phosphate | 56-73-5 |
| | F6P | Fructose 6-phosphate | 643-13-0 |
| | F1,6BP | Fructose 1,6-bisphosphate | 488-69-7 |
| | 2PG | 2-Phosphoglyceric acid | 2553-59-5 |
| | 3PG | 3-Phosphoglyceric acid | 820-11-1 |
| | PEP | Phosphoenolpyruvic acid | 138-08-9 |
| | Pyr | Pyruvic acid | 127-17-3 |
| PPP | 6PG | 6-Phosphogluconic acid | 921-62-0 |
| | R5P | Ribose 5-phosphate | 3615-55-2 |
| | S7P | Sedoheptulose 7-phosphate | 2646-35-7 |
| Other sugar phosphates | F1P | Fructose 1-phosphate | 15978-08-2 |
| | G1P | Glucose 1-phosphate | 59-56-3 |
| | M1P | Mannose 1-phosphate | 27251-84-9 |
| | GA6P | Glucosamine 6-phosphate | 3616-42-0 |
| | GAL1P | Galactose 1-phosphate | 2255-14-3 |
| | GL3P | Glycerol 3-phosphate | 17989-41-2 |
| | M6P | Mannose 6-phosphate | 3672-15-9 |
| | UDP-Glc-Nac | Uridine diphosphate-N-acetylglucosamine | 528-04-1 |
| | PRPP | Phosphoribosyl pyrophosphate | 7540-64-9 |
| TCA cycle | Cit | Citric acid | 77-92-9 |
| | ICit | Isocitric acid | 320-77-4 |
| | aKG | a-Ketoglutaric acid | 328-50-7 |
| | Suc | Succinic acid | 110-15-6 |
| | Fum | Fumaric acid | 110-17-8 |
| | Mal | Malic acid | 97-67-6 |
| | Lac | Lactic acid | 79-33-4 |
| Nucleoside phosphates | AMP | Adenosine monophosphate | 61-19-8 |
| | ADP | Adenosine diphosphate | 58-64-0 |
| | ATP | Adenosine triphosphate | 56-65-5 |
| | GMP | Guanosine monophosphate | 85-32-5 |
| | GDP | Guanosine diphosphate | 146-91-8 |
| | GTP | Guanosine triphosphate | 86-01-1 |
| | CMP | Cytidine monophosphate | 63-37-6 |
| | CDP | Cytidine diphosphate | 63-38-7 |
| | CTP | Cytidine triphosphate | 65-47-4 |
| | UMP | Uridine monophosphate | 58-97-9 |
| | UDP | Uridine diphosphate | 58-98-0 |
| | UTP | Uridine triphosphate | 63-39-8 |
| | IMP | Inosine monophosphate | 131-99-7 |
| | ITP | Inosine triphosphate | 132-06-9 |
| Amino acids | Ala | Alanine | 56-41-7 |
| | Arg | Arginine | 74-79-3 |
| | Asn | Asparagine | 70-47-3 |
| | Asp | Aspartic acid | 56-84-8 |
| | Cys | Cysteine | 52-90-4 |
| | Gln | Glutamine | 56-85-9 |
| | Glu | Glutamic acid | 56-86-0 |
| | Gly | Glycine | 56-40-6 |
| | His | Histidine | 71-00-1 |
| | Ile | Isoleucine | 73-32-5 |
| | Leu | Leucine | 61-90-5 |
| | Lys | Lysine | 56-87-1 |
| | Met | Methionine | 63-68-3 |
| | Phe | Phenylalanine | 63-91-2 |
| | Pro | Proline | 147-85-3 |
| | Ser | Serine | 56-45-1 |
| | Thr | Threonine | 72-19-5 |
| | Trp | Tryptophan | 73-22-3 |
| | Tyr | Tyrosine | 60-18-4 |
| | Val | Valine | 72-18-4 |
