# Supplementary material for: Survival of Escherichia coli after high-antibiotic stress is dependent on both the pregrown physiological state and incubation conditions
Source: Front Microbiol. 2023 Mar 10;14:1149978. doi: 10.3389/fmicb.2023.1149978 (PMC10036391; doi:10.3389/fmicb.2023.1149978)
Supplement: Supplementary file 3 [file Data_Sheet_3.PDF]

Supplementary Material

Survival of *Escherichia coli* after high-antibiotic stress is dependent on both the pregrown physiological state and incubation conditions

Lilja Brekke Thorfinnsdottir, Gaute Hovde Bø, James Alexander Booth, and Per Bruheim*

*** Correspondence:** Per Bruheim: per.bruheim@ntnu.no

1 Supplementary Figures

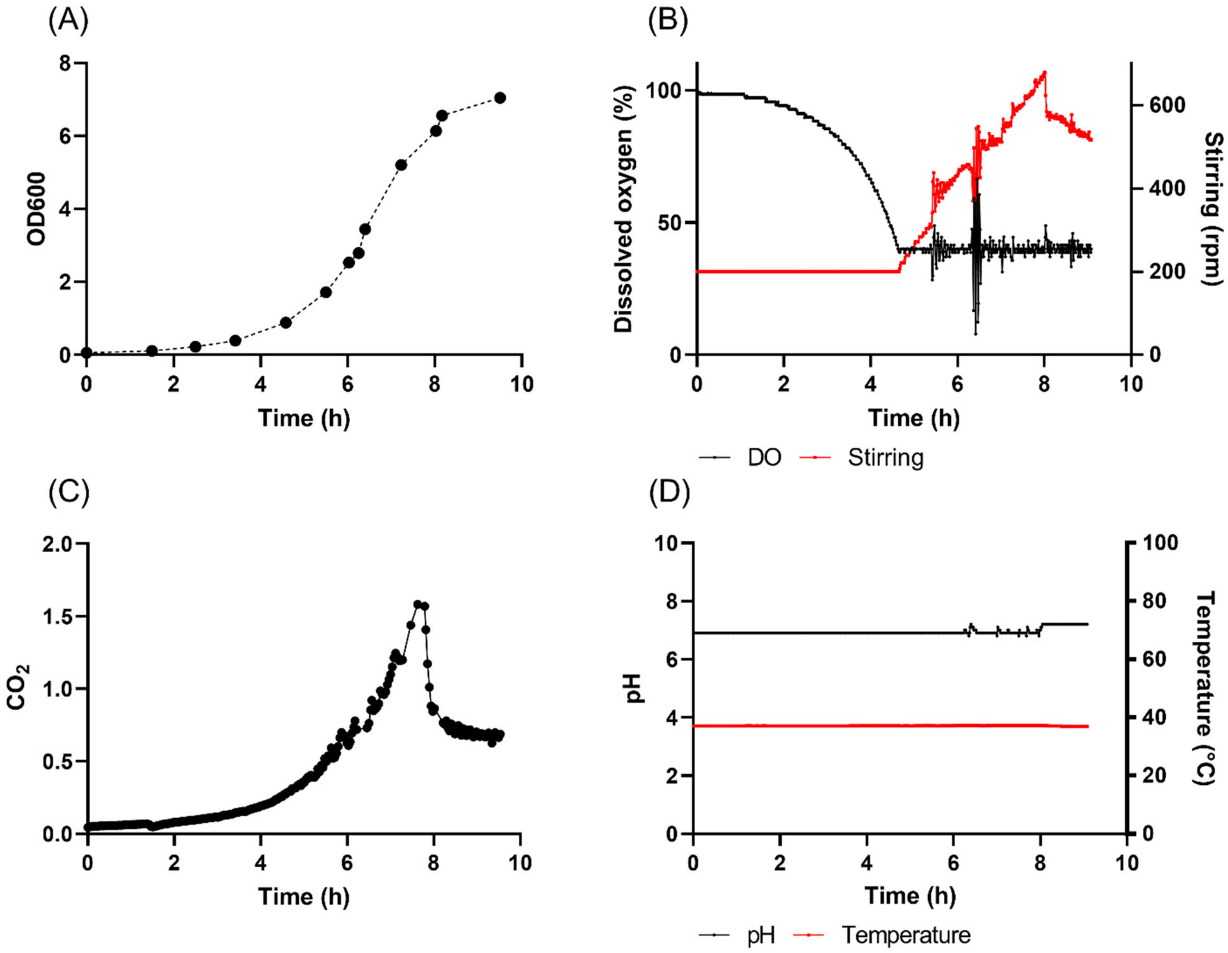

Supplementary Figure 1. Example data from a representative cultivation in the bioreactors. **(A)** Optical density measured at 600 nm, **(B)** dissolved oxygen (DO) and stirring, **(C)** offgas measurement of CO_2, and **(D)** pH and temperature.

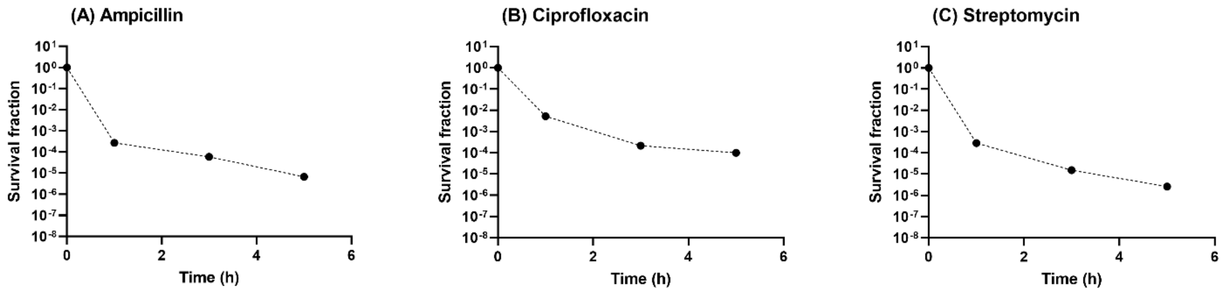

Supplementary Figure 2. Representative biphasic killing curves. Mid-exponential phase *E. coli* WT in shake flasks were treated with **(A)** 100 µg/mL ampicillin, **(B)** 5 µg/mL ciprofloxacin, or **(C)** 25 µg/mL streptomycin for 5 h. Viable cell counts were assayed at 0, 1, 3, and 5 h after treatment, and the surviving fractions were calculated. One representative experiment out of five is presented.

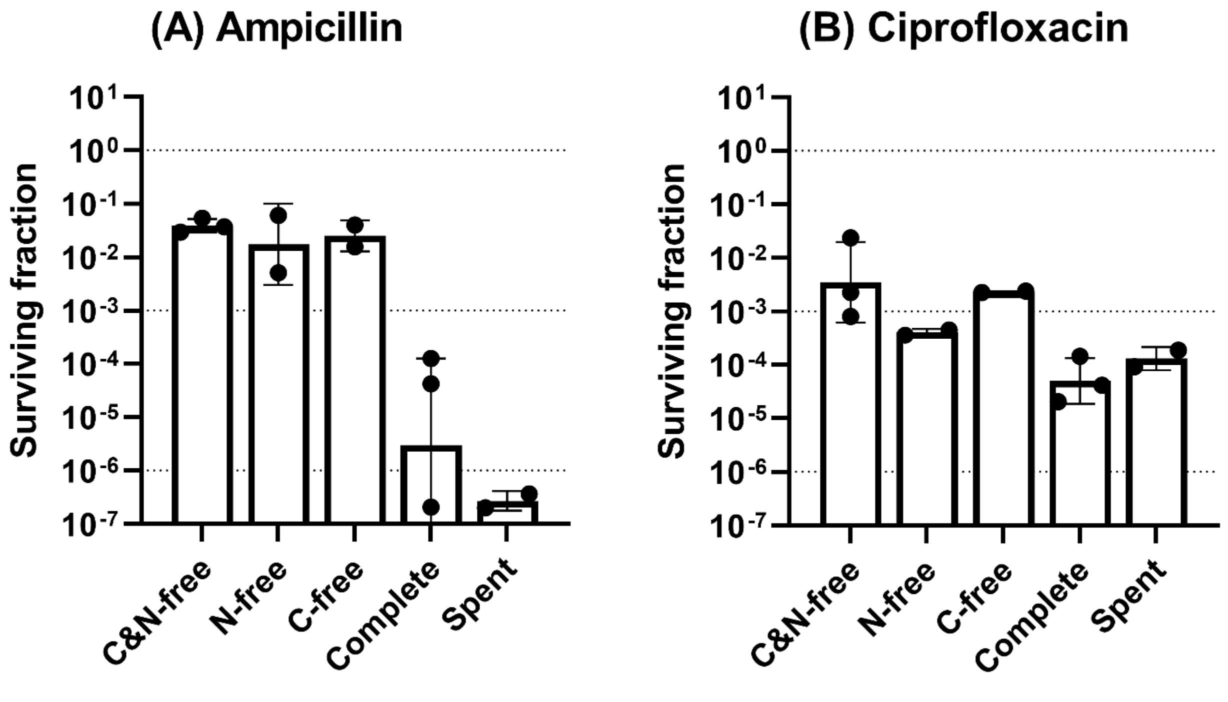

Supplementary Figure 3. Surviving fractions after HAS in different media. Aliquots of mid-exponential phase *E. coli* WT at OD600 = 0.5 in shake flasks were centrifuged and resuspended in different stress media (without either carbon or nitrogen source or both, complete, or spent) and treated with **(A)** 100 µg/mL ampicillin or **(B)** 5 µg/mL ciprofloxacin for 5 h. Viable cell counts were assayed at 0 and 5 h after treatment, and the surviving fractions at 5 h were calculated. Geometric mean and geometric standard deviation from 2-4 experiments are plotted, with individual experiments presented as dots. Dotted lines at 10^0, 10^{-3}, and 10^{-6} are added to aid interpretation. Values from individual experiments are added HAS, high-antibiotic stress; C&N-free, lacking both

carbon and nitrogen; N-free, lacking nitrogen; C-free, lacking carbon; spent, supernatant from mid-exponential phase cultures.

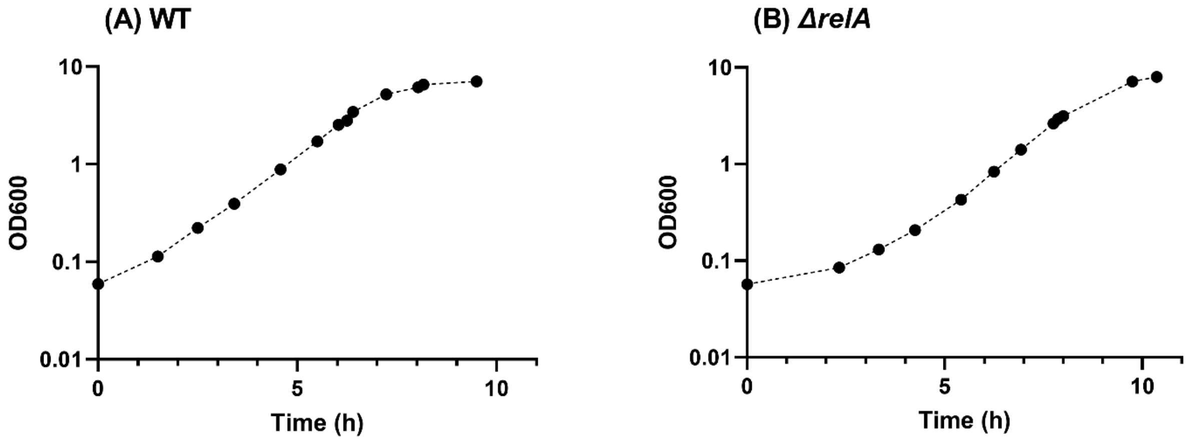

Supplementary Figure 4. Growth curves for **(A)** *E. coli* WT and the **(B)** *ΔrelA* mutant from one representative experiment. The *ΔrelA* mutant has a longer lag time, but the strains have the same growth rate (0.65-0.75 h^{-1}), hence a generation time of 0.9-1.1 h^{-1}.

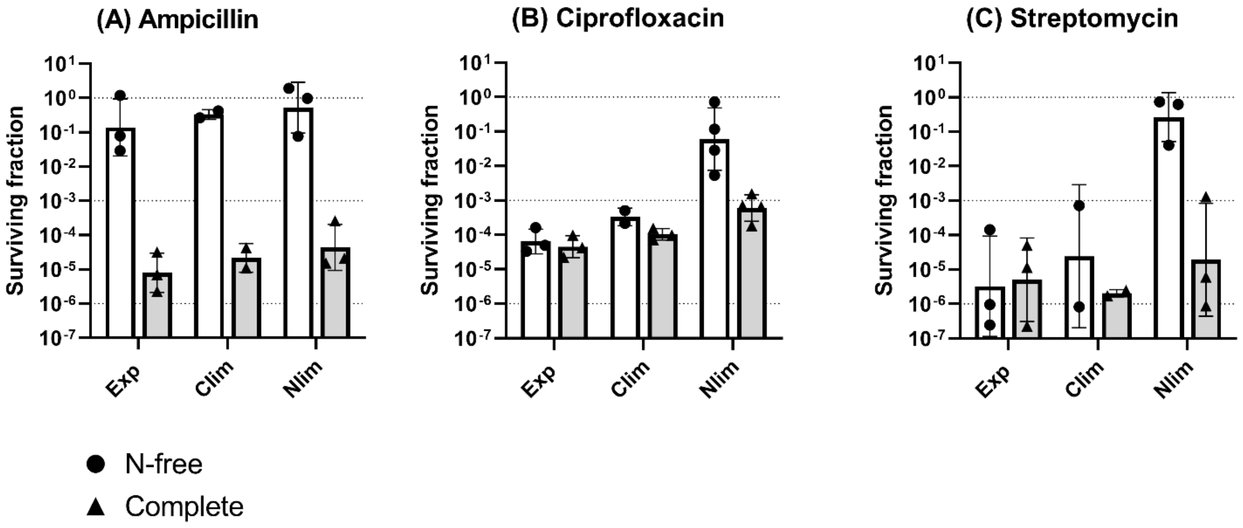

Supplementary Figure 5. Survival of *E. coli* WT cells pregrown to three physiological states and treated with antibiotics in two stress media. *E. coli* WT cells were pregrown to three physiological states in benchtop bioreactors before centrifugation and resuspension into two stress media with **(A)** 100 µg/mL ampicillin, **(B)** 5 µg/mL ciprofloxacin or **(C)** 25 µg/mL streptomycin. Viable cell counts were assayed at 0 and 5 h after treatment, and the surviving fractions at 5 h were calculated. Geometric mean and geometric standard deviation from 2-4 experiments are plotted, with individual experiments presented as dots. Dotted lines at 10^0, 10^{-3}, and 10^{-6} are added to aid interpretation. N-

free, HAS medium lacking nitrogen; Exp, exponential phase; Clim, carbon-limited stationary phase; Nlim, nitrogen-limited stationary phase.

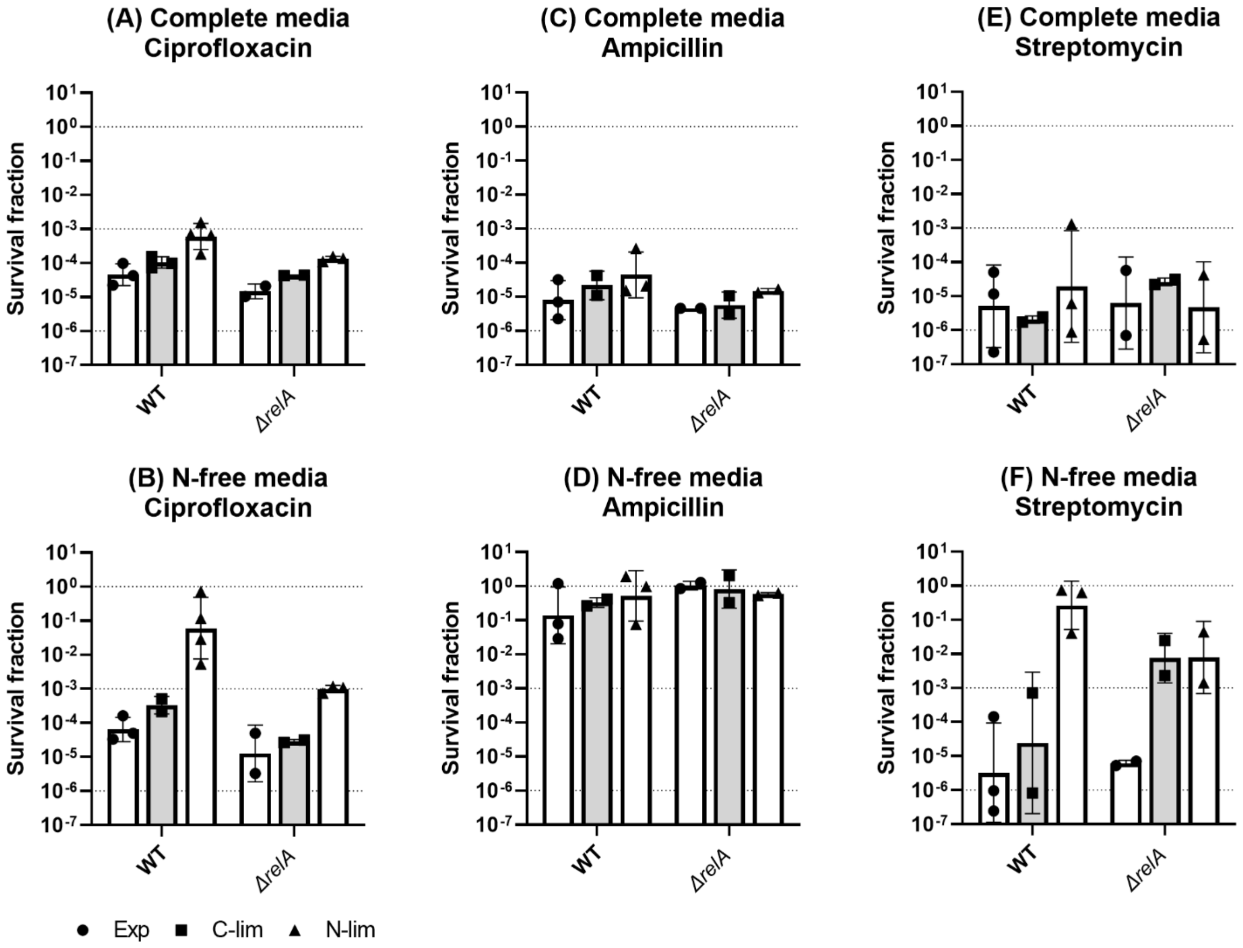

Supplementary Figure 6. Survival of *E. coli* WT and *ΔrelA* cells**.** *E. coli* WT and *ΔrelA* cells were pregrown to three physiological states (exponential phase, carbon or nitrogen-limited stationary phase; Clim or Nlim) in benchtop bioreactors, before centrifugation and resuspension into two stress media with **(A)** and **(B)** 100 µg/mL ampicillin, **(C)** and **(D)** 5 µg/mL ciprofloxacin or **(E)** and **(F)** 25 µg/mL streptomycin. Viable cell counts were assayed at 0 and 5 h after treatment, and the surviving fractions at 5 h were calculated. Geometric mean and geometric standard deviation from 2-4 experiments are plotted, with individual experiments presented as dots. Dotted lines at 10^0, 10^{-3}, and 10^{-6} are added to aid interpretation. WT results from Figure 2 are included to ease comparison and interpretation. N-free, HAS medium lacking nitrogen; Exp, exponential phase; Clim, carbon-limited stationary phase; Nlim, nitrogen-limited stationary phase.

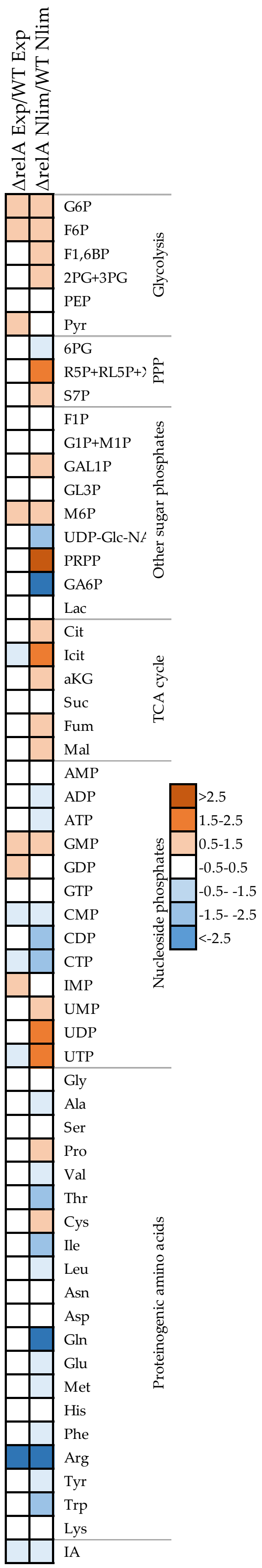

Supplementary figure 7: Log_2 fold differences between *E. coli* WT and the *ΔrelA* mutant in exponential (left) and Nlim stationary phase (right).
